# Computational prediction and characterisation of miRNAs and their pathway genes in human schistosomiasis caused by *Schistosoma haematobium*

**DOI:** 10.1590/0074-02760190378

**Published:** 2020-05-08

**Authors:** Thaís Cunha de Sousa Cardoso, Carlos Bruno de Araújo, Laysa Gomes Portilho, Luiz Guilherme Alves Mendes, Tamires Caixeta Alves, Gustavo Caetano Silva, Thales Henrique Cherubino Ribeiro, Peterson Elizandro Gandolfi, Enyara Rezende Morais, Laurence Rodrigues do Amaral, Matheus de Souza Gomes

**Affiliations:** 1 Universidade Federal de Uberlândia, Laboratório de Bioinformática e Análises Moleculares, Patos de Minas, MG, Brasil; 2 Universidade Federal de Lavras, Departamento de Biologia, Setor de Fisiologia Vegetal, Laboratório de Fisiologia Molecular de Plantas, Lavras, MG, Brasil; 3 Universidade Federal de Uberlândia, Rede Multidisciplinar de Pesquisa, Ciência e Tecnologia, Patos de Minas, MG, Brasil

**Keywords:** human parasite, small RNAs, bioinformatics, neglected diseases

## Abstract

**BACKGROUND:**

Key genes control the infectivity of the *Schistosoma haematobium* causing schistosomiasis. A method for understanding the regulation of these genes might help in developing new disease strategies to control schistosomiasis, such as the silencing mediated by microRNAs (miRNAs). The miRNAs have been studied in schistosome species and they play important roles in the post-transcriptional regulation of genes, and in parasite-host interactions. However, genome-wide identification and characterisation of novel miRNAs and their pathway genes and their gene expression have not been explored deeply in the genome and transcriptome of *S. haematobium*.

**OBJECTIVES:**

Identify and characterise mature and precursor miRNAs and their pathway genes in the *S. haematobium* genome.

**METHODS:**

Computational prediction and characterisation of miRNAs and genes involved in miRNA pathway from *S. haematobium* genome on SchistoDB. Conserved domain analysis was performed using PFAM and CDD databases. A robust algorithm was applied to identify mature miRNAs and their precursors. The characterisation of the precursor miRNAs was performed using RNAfold, RNAalifold and Perl scripts.

**FINDINGS:**

We identified and characterised 14 putative proteins involved in miRNA pathway including ARGONAUTE and DICER in *S. haematobium*. Besides that, 149 mature miRNAs and 131 precursor miRNAs were identified in the genome including novel miRNAs.

**MAIN CONCLUSIONS:**

miRNA pathway occurs in the *S. haematobium*, including endogenous miRNAs and miRNA pathway components, suggesting a role of this type of non-coding RNAs in gene regulation in the parasite. The results found in this work will open up a new avenue for studying miRNAs in the *S. haematobium* biology in helping to understand the mechanism of gene silencing in the human parasite *Schistosome*.

The human schistosomiasis is a parasitic, chronic and debilitating disease caused by helminths of the *Schistosoma* genus that currently affects about 240 million people worldwide, according to the World Health Organization.^1^ The countries of Africa and South America are among the most affected. In addition, 800 million people live in areas at risk of contracting the disease.^2^ Five *Schistosoma* species are responsible for most of human infections: *Schistosoma mansoni*, *Schistosoma japonicum*, *S. haematobium*, *Schistosoma mekongi* and *Schistosoma intercalatum*.^1^

*S. haematobium* affects the reproductive and urinary system and it has been considered a major public health problem mainly in Africa.^1^ ^,^ ^3^ The infection by this parasite can results in nutritional deficiencies and growth retardation, may also lead to a decrease in the cognitive system.^4^ The spread of this disease occurs by fresh water and by the presence of host snails. Activities in water contact, such as agriculture, can facilitate the parasite infection.^3^

The dissemination of the disease is dependent of interaction between parasite and intermediate host. In *S. mansoni* and their intermediate host *Biomphalaria glabrata*, the interaction is complex and determined by some genes involved in the parasite infectivity and host susceptibility.^5^ ^,^ ^6^ Thus, it is also suggested a complex host/parasite interation involving several genes between *S. haematobium* and its intermediate snail host, from the genus *Bulinus*. Therefore, understanding the mechanisms of gene regulation is critically important for understanding the host-parasite interactions and which genes might contribute to the infection.

The regulation of gene expression in eukaryotes comprises processes involved in translation repression and/or degradation of target genes.^7^ Small RNAs, such as microRNAs (miRNAs), and their silencing pathways have been considered important in several organisms by performing a fine and specific regulation of gene expression maintaining genome integrity.^7^ ^,^ ^8^

The miRNAs are small non-coding endogenous RNAs with about 22 nucleotides in length of acting post-transcriptionally typically via imperfect complementary base pairing binding to the target genes.^7^ ^,^ ^9^ ^,^ ^10^ To identify mature miRNAs and their precursors several studies have been using experimental and computational strategies. The experimental approaches, despite presenting physical evidence of the miRNAs presence, can exclude molecules expressed in certain stages and tissues.^10^ ^,^ ^11^ ^,^ ^12^ Thus, *in silico* analysis are useful for species with complete or incomplete sequenced genomes, allowing the discovery of new miRNAs using whole-genome DNA information.^11^

The genes involved in gene silencing pathway mediated by miRNAs have been described in several model organisms such as *Drosophila melanogaster*, *Caenorhabditis elegans* and *Homo sapiens*.^13^ ^,^ ^14^ In addition, members of the biosynthetic pathway of miRNAs, such as ARGONAUTE and DICER, as well as miRNA molecules, have been reported in some *Schistosoma* species, including *S. mansoni*, *S. japonicum* and *S. haematobium*.^15^

Young et al., published in 2012 the whole-genome sequence of *S. haematobium*.^16^ The interpretation of schistosome draft genomes has improved our understanding of the molecular biology of these parasites allowing the identification and characterisation of undiscovered genes. In the face of the vastly increased importance of such non-coding RNAs in the gene expression regulation, it is important that we study mature and precursor miRNAs, as well as the genes involved in miRNA pathway in this *S. haematobium*. This might help to understand the life cycle of this parasite, its infectivity mechanism and for searching new methods of schistosomiasis control. In this work, we identified and characterised the miRNA pathway genes, the mature miRNAs and their precursors in the *S. haematobium* genome. The results found in this work will open up a new avenue for studying miRNAs in the *S. haematobium* biology in helping to understand the mechanism of gene silencing in the human parasite *Schistosome*.

## MATERIALS AND METHODS

*Prediction and characterisation of genes involved in the miRNAs pathway* - Firstly, to search the putative protein sequences involved in the miRNA pathway in *Schistosoma haematobium*, BLASTp tool was used (National Centre for Biotechnology Information ― http://www.ncbi.nlm.nih.gov/) using as queries sequence reference proteins from animal species, such as *D. melanogaster* and *C. elegans*, model organisms. The putative protein sequences in *S. haematobium* were found and collected from SchistoDB, *S. haematobium* Egypt genome (v 44, 2019-06-24) (http://schistodb.net). To improve the annotation of these predicted proteins involved in the miRNA pathway in *S. haematobium*, other gene prediction technique was used. The *S. haematobium* Egypt genome (v 44, 2019-06-24) was retrieved from the *Schistosoma* Genomic Resource database. To improve gene prediction of the putative genes involved in miRNA pathway in *S. haematobium* genome, single-end reads from a single cDNA library of *S. mansoni* were retrieved from SRA accession SRR629229. After quality control, approximately 63% of 36.836.649 reads were successfully mapped to the *S. haematobium* Egypt genome with STAR (v 2.5.3ab) using the parameter twopassMode.^17^ The introns coordinates from this alignment were converted to GFF format to train GeneMark-ET algorithm (v 4.33).^18^ In this way 19.686 putative genes were predicted. In addition, GeneMark-ES (v 4.33) was also applied to *S. haematobium* Egypt genome to predict genes without RNA-seq support, 18.622 putative genes were predicted with this approach. To identify the orthologous genes, BLAT (v 35) was applied to search query proteins against the reference genome.^19^ The top 10 hits of each query were manually compared against the two sets of predicted genes using the Integrated Genome Browser (v 9.0.0).^20^ In addition, Augustus web tool (http://augustus.gobics.de/) was used to improve gene prediction using the genomic locus around the BLAT results, setting the parameters to find similarities with *S. mansoni* genes. The consensus sequences, from the BLAT alignment, and the predicted genes were then aligned to their orthologous using CLUSTALX (v 2.1) (http://www.clustal.org/) and finally subjected to conserved domains searches with PFAM online tool (v 31.0) (http://pfam.xfam.org/).

The characterisation and *in silico* validation of the putative protein sequences involved in the miRNA pathway in *S. haematobium* were performed using the position and the presence of the conserved domains and also of the amino acids from active sites using PFAM and Conserved Domains Database (CDD) (http://www.ncbi.nlm.nih.gov/cdd/).

*Expression analysis* - The transcripts of the genes involved in the miRNA pathway in *S. haematobium* were individually analysed using RNASeq data set, retrieved from NCBI Sequence Read Archive ― SRA, of three different developmental stages of *S. haematobium*: adult female (SRR6655495), adult male (SRR6655497) and egg (SRR6655493).^16^ Quality control and adapter removal were conducted using Trimmomatic (v 0.36). Single-end reads were aligned against pre-selected sequences using bowtie2 (v 2.3.0) with the “--very-sensitive-local parameter”. Alignment sam files were sorted and converted to bam files with samtools (v 1.6). Expression values were extracted from the alignment results using express (v 1.5.1) and RPKM (Reads Per Kilobase of transcript, per Million mapped reads) were calculated after library size normalisation with the Bioconductor package edgeR in the R statistical environment. The results of the expression analysis were plotted in a heatmap, where the colour system applied comprises an intense red colour as the highest transcript expression and an intense blue colour as the lowest transcript expression.

*Prediction and characterisation of mature miRNAs and their precursors (pre-miRNAs)* - A robust algorithm used to predict miRNAs in *S. mansoni* genome^10^ was also used for identification and characterisation of mature miRNAs and their precursors in *S. haematobium* Egypt genome*.* The data was applied to Einverted (EMBOSS tool)^21^ and BLASTN programs (http://www.ncbi.nlm.nih.gov/) to select sequences that show potential hairpin formation or similarities with precursor miRNAs. These sequences were applied in a set of filters accepting in the end of the algorithm putative real conserved miRNAs. These filters were based on conserved characteristics of precursor miRNAs and regions that have no potential formation of pre-miRNAs were discarded. The filters used were: guanine and cytosine (GC content), minimal energy free (MFE), homology with mature miRNAs already identified, homology with protein coding region, homology to repetitive regions and homology with non-coding RNAs.

The miRNA cluster searching was optimised using information obtained from the position (start and the end position of the precursor miRNAs) each miRNA identified in the *S. haematobium* Egypt genome. miRNAs found at a distance of less than 10k nucleotides downstream or upstream depending on the direction in the DNA strand were considered linked in a specific miRNA cluster. Each sequence portion was retrieved from the *S. haematobium* Egypt genome using information from the first and last nucleotides of the cluster. The secondary structure of each cluster was obtained using the RNAfold tool (http://rna.tbi.univie.ac.at/cgi-bin/RNAWebSuite/RNAfold.cgi).

miRNA sequences identified as putative real conserved miRNA precursors in *S. haematobium* genome were characterised structurally and thermodynamically using RNAfold, RNAalifold (Vienna RNA Package) (http://rna.tbi.univie.ac.at/cgi-bin/RNAWebSuite/RNAalifold.cgi) and homemade Perl scripts.^22^

*miRNAs in S. haematobium Egypt small RNA-seq libraries* - Two small RNA data files of adult male (SRR6655496) and adult female worms (SRR6655494) were retrieved from the NCBI Sequence Read Archive (SRA). The library qualities were evaluated using FastQC software.^23^ The adapters were removed with Trimmomatic^24^ discarding reads with quality score below 20 and length less than 17 nucleotides and longer than 30 nucleotides. The filtered sequences were mapped and quantified using miRDeep2.^25^ miRDeep2 and perl scripts were used on each sequence separately to generate the numbers of the reads for each miRNAs identified.

*Sequence alignments and phylogenetic analysis* - The multiple sequence alignment (MSA) were performed to *S. haematobium* Egypt putative proteins involved in the miRNA pathway and also for the *S. haematobium* Egypt pre-miRNA sequences and their respective orthologs. These analyses were performed by CLUSTALX 2.0 with default parameters multiple sequence alignment of proteins and for the pre-miRNA sequences were used the following adjusted parameters: gap opening 22.50 and gap extension 0.83.

Phylogenetic analysis was performed using MEGA version X^26^ and Neighbor-Joining.^27^ A consensus tree was generated applying a bootstrap of 5.000 replicates for pre-miRNA sequences and 2.000 replicates for protein sequences. The calculation of their evolutionary distance was performed using Kimura-2-parameters for the pre-miRNA and JTT for protein sequences.^27^

*Statistical analysis* - For the statistical comparisons among the structural and thermodynamic variables of each category (superphylum, phylum and/or genus), a basic descriptive analysis was performed followed by non-parametric tests (Wilcoxon-Mann-Whitney). Median values were used to perform the statistical comparisons.^28^ Statistical significance was set at p < 0.05.

## RESULTS

*Identification, characterisation, phylogenetic analysis of putative proteins involved in miRNA pathway* - The predicted proteome version 44 of the *S. haematobium* genome obtained from SchistoDB was used to identify and characterise the predicted *S. haematobium* proteins involved in the miRNA pathway. Fourteen putative *S. haematobium* proteins involved in the miRNA pathway were identified and characterised according to their putative function based on homology, conserved domain distribution (and conservation) and protein size. These *S. haematobium* proteins were compared with their miRNA pathway orthologue proteins from related species such as *Schistosoma* species and model species (e.g. *C. elegans* and *D. melanogaster*) (Table). In order to confirm and improve the prediction of the proteome version 44 of the *S. haematobium* an alternative predicted genome-based proteome was performed using information from the *S. haematobium* genome and RNAseq sequences (SRR6655495, SRR6655497, SRR6655493). 18.622 putative genes were predicted with this alternative approach to predict the *S. haematobium* proteome. Comparing the proteins involved in the miRNA pathway predicted from *S. haematobium* version 44 to proteins obtained from the alternative predicted proteome only the DROSHA protein sequence was divergent. The putative DROSHA sequence predicted from proteome version 44 - SchistoDB showed 1041 amino acids in length and only one Ribonuclease III conserved domain. The new prediction of DROSHA showed a protein with two Ribonuclease III and one DSRM conserved domains with 1581 amino acids in length corroborating with their orthologs and thus used for further analysis. Among the fourteen putative *S. haematobium* proteins involved in the miRNA pathway identified, two key protein families of the canonical miRNA biogenesis pathway were considered for the further analysis: ARGONAUTE and RNAse III proteins (DICER and DROSHA). We were able to identify and characterise in *S. haematobium* data and considered for the further analysis two AGO proteins (sht_Ago_MS3_08447/ sht_Ago_MS3_01142), two DICER proteins (sht_Dcr_MS3_05083 and sht_Dcr_MS3_09247) and one DROSHA protein (sht_Drsh_MS3_06910).

**TABLE:** Putative proteins involved in miRNA pathway identified in *Schistosoma haematobium* data compared with their orthologs from *Schistosoma mansoni* and *Schistosoma japonicum*

ID protein *S. haematobium*	Putative name	Length (aa)	ID protein *S. mansoni*	E-value	Length (aa)	ID protein *S. japonicum*	E-value	Length (aa)
MS3_01142	Sht_Argonaute	1009	Smp_198380.1	0.0	928	Sjp_0044720	0.0	987
			Smp_102690.1	4 e^-105^	783	Sjp_0103990	2 e^-106^	904
			Smp_179320.2	1 e^-118^	921	Sjp_0045200	2 e^-115^	924
MS3_08447	Sht_Argonaute	882	Smp_198380.1	5 e^-101^	928	Sjp_0044720	7 e^-101^	987
			Smp_102690.1	0.0	783	Sjp_0103990	0.0	904
			Smp_179320.2	0.0	921	Sjp_0045200	0.0	924
MS3_06910	Sht_Drosha	1581	Smp_142510.1	0.0	1531	Sjp_0048900.1	0.0	1611
MS3_05083	Sht_Dicer	929	Smp_169750.2	0.0	2485	Sjp_0069770	4.2 e^-129^	2480
			XP_018644375.1	0.0	2319	-	-	-
MS3_09247	Sht_Dicer	2588	Smp_033600	5 e^-17^	954	Sjp_0043700	2e^-17^	923
MS3_10205	Sht_Exportin 5	611	Smp_137650	0.0	1164	Sjp_0006510	0.0	695
MS3_11315	Sht_TSN	1162	Smp_246840	8.4 e^-116^	992	Sjp_0048060	5 e^-165^	1428
MS3_03052	Sht_Logs	284	Smp_023670	0.0	356	Sjp_0045700	6 e^-178^	369
MS3_05484	Sht_FRX1	339	Smp_099630	0.0	598	Sjp_0017160	0.0	598
MS3_06671	Sht_Exportin-1	1031	Smp_124820	0.0	828	Sjp_0069890	0.0	1128
MS3_07205	Sht_TSN-a	718	Smp_166110	8 e^-82^	378	Sjp_0100020	0.0	971
MS3_08368	Sht_DGCR8	298	Smp_087220	0.0	760	Sjp_0013270	0.0	760
MS3_10949	Sht_PASHA	395	Smp_087220	0.0	760	Sjp_0013270	2 e^-129^	760
MS3_11239	Sht_VIG	237	Smp_009310	2 e^-121^	417	Sjp_0074950	5 e^-97^	414

**Fig. 1:**
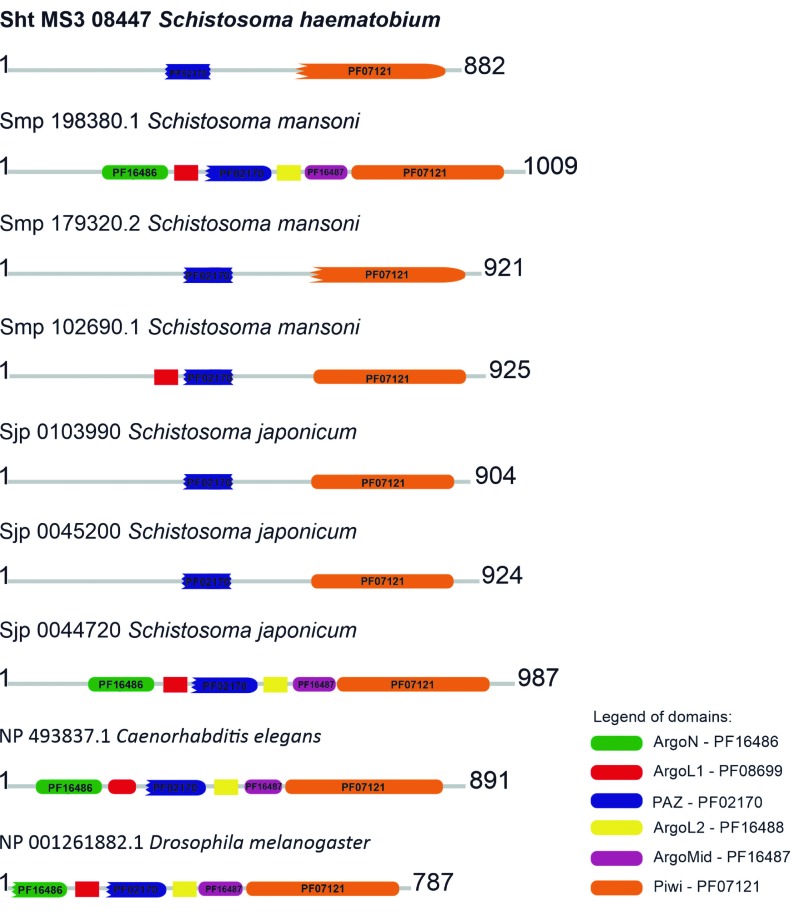
conserved domains found in AGO proteins of *Schistosoma haematobium* Egypt versus their orthologous from *S. mansoni* and *S. japonicum*, and model organisms *Caenorhabditis elegans* and *Drosophila melanogaster*.

*ARGONAUTE* - The putative sht_Ago_MS3_08447 protein displayed only the PIWI and PAZ domains at positions 559-852 and 304-400, respectively. On the other hand, the putative protein sht_Ago_MS3_01142 showed the PAZ, PIWI, ARGOL1, ARGOL2, ARGON and ARGOMID domains. These *S. haematobium* Ago proteins and the distribution of their conserved domains corroborated with their orthologues in *S. mansoni* (Smp) and *S. japonicum* (Sjp)^15^ (Fig. 1).

**Fig. 2:**
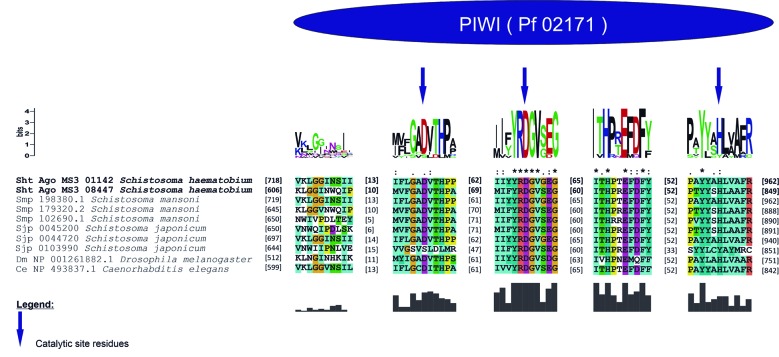
analysis of PIWI conserved domain of *Schistosoma haematobium* AGO proteins.

The PIWI domain of these proteins showed a catalytic triad formed by DDH (aspartic acid, aspartic acid and histidine). In sht_Ago_MS3_08447, the catalytic triad was found at positions Asp621, Asp711 and His844. In sht_Ago_MS3_01142, the same triad was identified at positions Asp747, Asp819 and His957. Besides that, this triad showed high conservation in relation to ortholog AGO proteins (Fig. 2).

**Fig. 3:**
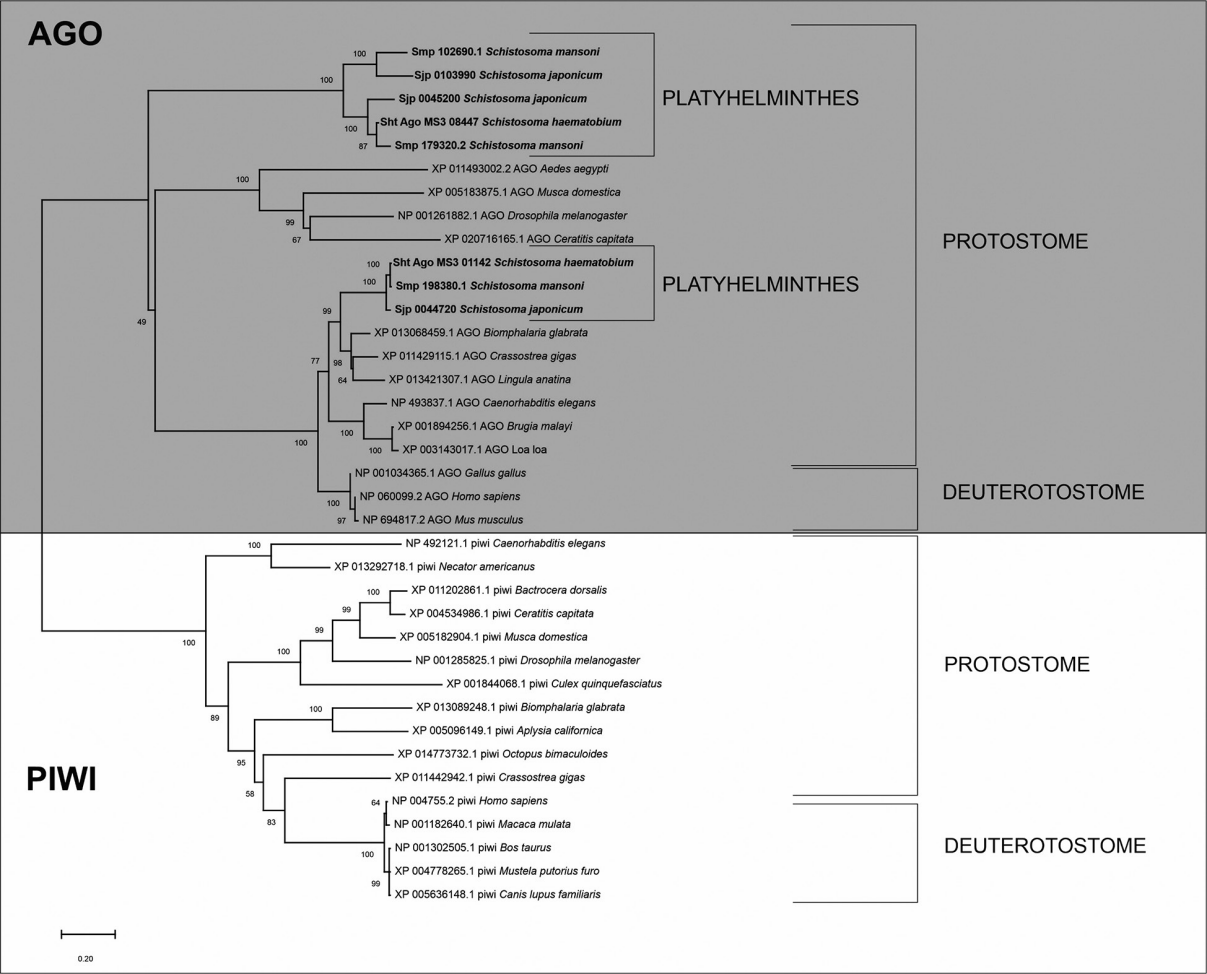
phylogenetic analysis of *Schistosoma haematobium* AGO proteins and their orthologous.

The AGO protein family can be divided into two subfamilies, AGO and PIWI. AGO and PIWI orthologous proteins from animal species were used to compare with the putative *S. haematobium* AGO proteins. The organisation of the AGO protein family clades in the phylogenetic analysis occurred as expected, in distinct clades, AGO subfamily grouped with AGO orthologous proteins including *S. haematobium* AGO proteins and PIWI subfamily with PIWI orthologous proteins. The distribution of the AGO and PIWI proteins from animal species corroborate with the species distribution in the tree of life (Fig. 3). We observed that *S. haematobium* putative proteins showed a suitable organisation compared with their orthologs, in the Plathyhelminthe clade, with *S. haematobium*, *S. mansoni and S. japonicum* proteins clustering closest within the clade.

**Fig. 4:**
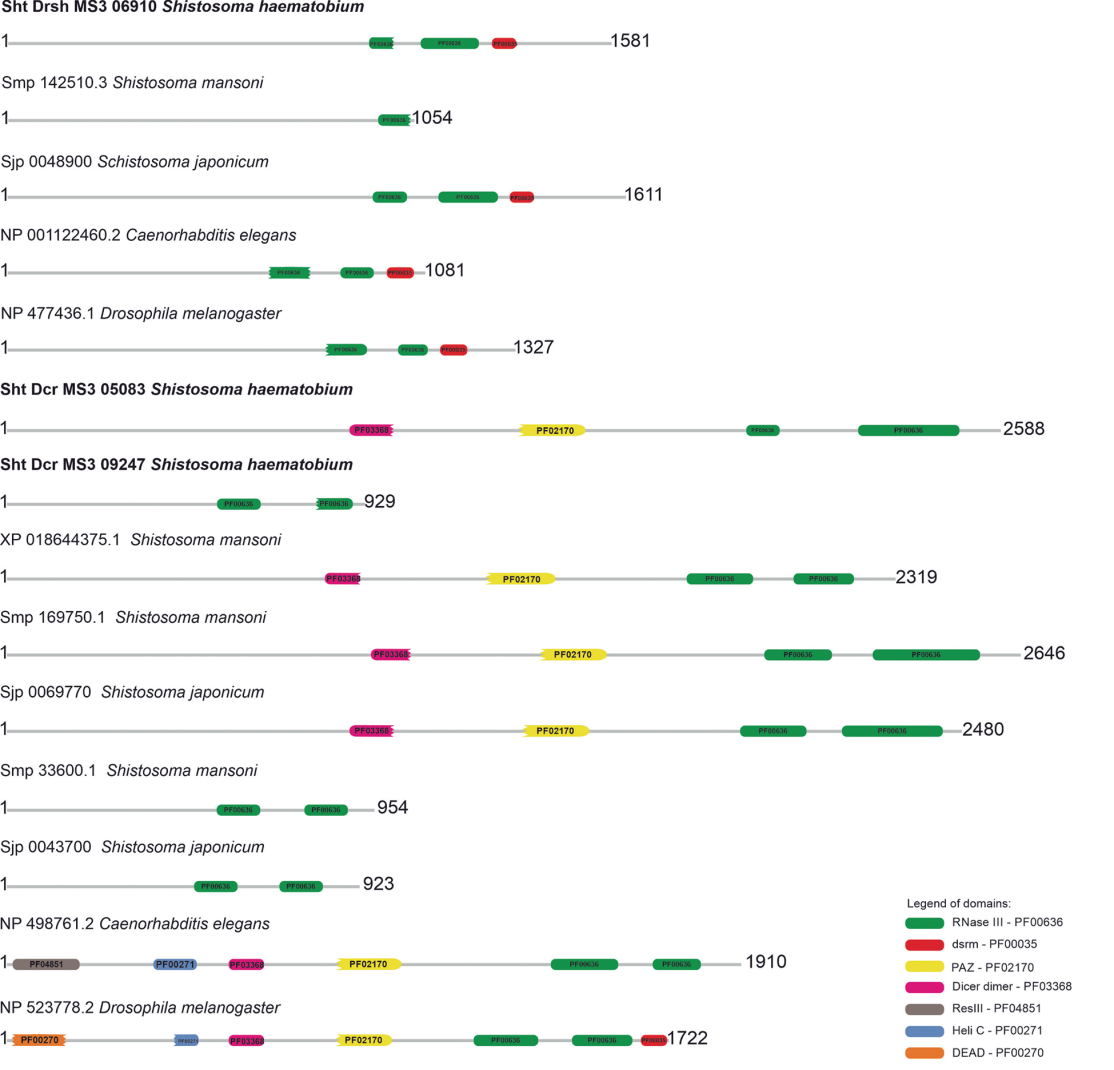
conserved domains of *Schistosoma haematobium* DICER/DROSHA proteins and their orthologous from *S. mansoni* and *S. japonicum*, and model organisms *Caenorhabditis elegans* and *Drosophila melanogaster.*

*DICER and DROSHA* - Two DICER proteins (sht_Dcr_MS3_05083 and sht_Dcr_MS3_09247) and one DROSHA protein (sht_Drsh_MS3_06910) were identified and characterised. The sht_Dcr_MS3_05083 displayed four domains, while sht_Dcr_MS3_09247 two conserved domains, and sht_Drsh_MS3_06910 three conserved domains. The putative sht_Dcr_MS3_05083 protein identified in the *S. haematobium* Egypt genome presented, in its structure, the conserved domains Dicer Dimer, PAZ, Ribonuclease III (Riboc I), Ribonuclease III (Riboc II), in the positions 896-1005, 1336-1508, 1926-2102, 2212-2474, respectively. The putative sht_Dcr_MS3_09247 presented only the Ribonuclease III (Riboc I) and Ribonuclease III (Riboc II) domains, in the positions 548-659 and 801-889. Finally, the putative sht_Drsh_MS3_06910 showed the Ribonuclease III (Riboc I), Ribonuclease III (Riboc II) and DSRM domains, at positions 941-1002, 1076-1229, 1258-1327, respectively, in its structure (Fig. 4).

**Fig. 5:**
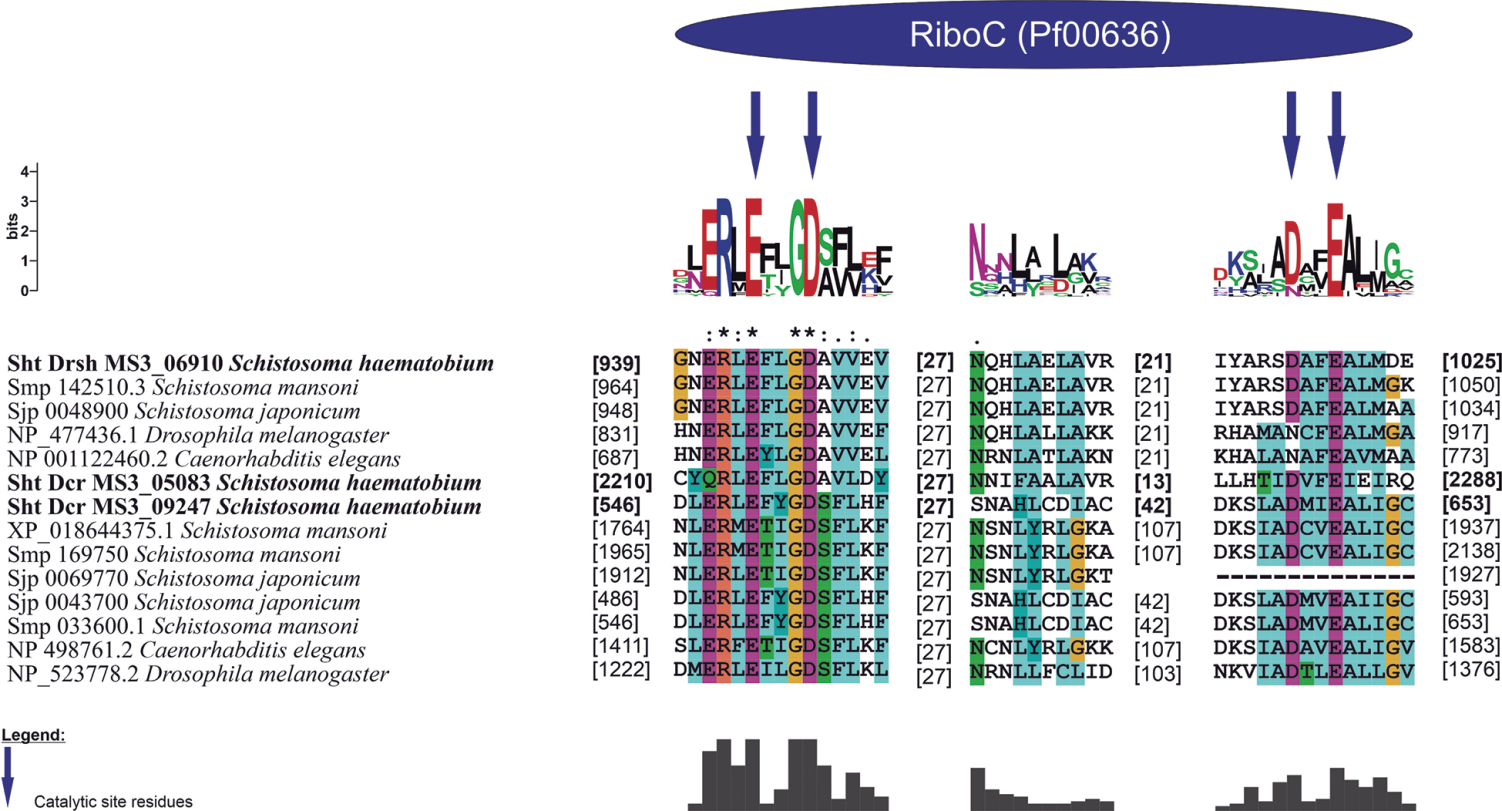
analysis of ribonuclease III (Riboc I and II) conserved domains of *Schistosoma haematobium* DICER and *S. haematobium* DROSHA proteins and their orthologous.

The two Ribonuclease III conserved domains identified in the putative DICER proteins (sht_Dcr_MS3_05083 and sht_Dcr_MS3_09247), showed an active site high conserved compared to their orthologs. Both domains displayed the conserved active site catalytic motif (EDDE): glutamic acid (E), aspartate (D), aspartate (D) and glutamic acid (E). The domains Riboc I in the sht_Dcr_MS3_05083 presented the catalytic residues conserved, at positions Glu2215, Asp2219, Asp2280 and Glu2283 and in sht_Dcr_MS3_09247 at positions Glu551, Asp555, Asp645 and Glu648. In the putative protein sht_Drs_MS3_06910 the catalytic residues of Riboc I domain showed in the positions Glu944, Asp948, Asp1017 and Glu1020 (Fig. 5).

**Fig. 6:**
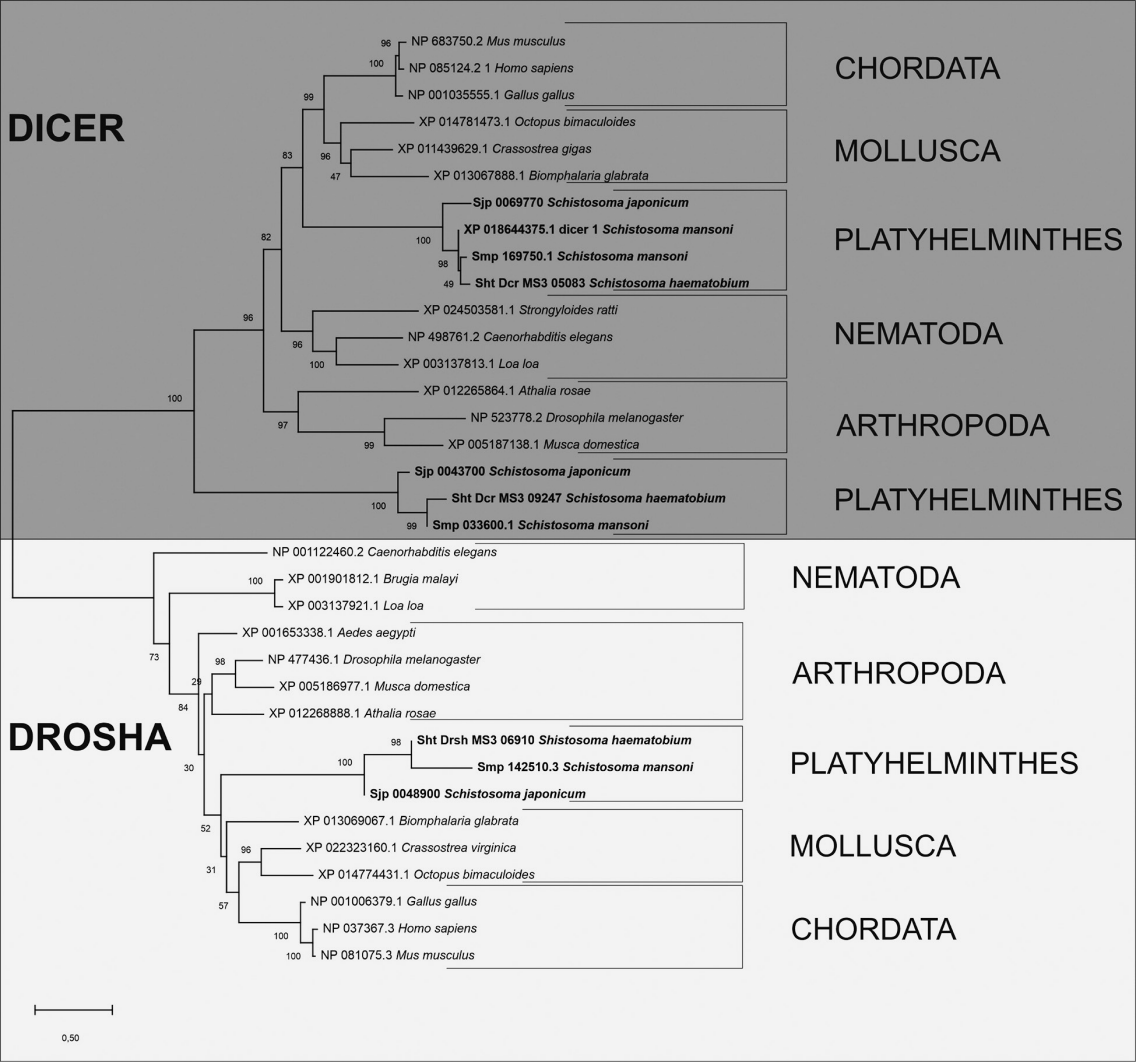
phylogenetic analysis of the *Schistosoma haematobium* DICER and *S. haematobium* DROSHA proteins and their orthologous.

The phylogenetic analysis of DICER and DROSHA putative proteins displayed the evolutionary relationship with their orthologs and paralogues. The *S. haematobium* proteins clustered within Platyhelminthes clade closest to *S. mansoni* and *S. japonicum* DICER and DROSHA proteins. The phylogenetic tree displayed two distinct clades (DICER and DROSHA clades) corroborating with the literature and the distribution of the species in the tree of life (Fig. 6).

*Expression analysis of miRNA pathway transcripts* - The genes involved in miRNA pathway identified in the genome data of *S. haematobium* were used to perform the expression analysis. This procedure occurred through submitting these transcripts individually to analyse against RNASeq data set for a library of three different stages of development in *S*. *haematobium*, adult female (SRR6655495), adult male (SRR6655497) and egg (SRR6655493). The heatmap showed the expression profile of the transcripts in the egg, adult female and adult male stages of the *S. haematobium* [Supplementary data I **(Fig. 1)**]. The gene that showed a highest expression level was the VIG (MS3_11239) that showed a more intense red colour in the heatmap for the three stages presented. The transcript of FMR1 (MS3_05484) also showed high level of expression in the three stages. The Expo1 transcript (MS3_06671) presented a high expression in egg and adult male, and a medium expression in adult female. The TSNA transcript (ID MS3_07205) presented a high expression in egg and adult female and a medium expression in adult male. The transcripts of the putative ARGONAUTE (sht_Ago_MS3_08447), showed a high level of expression in adult female and egg, and a low expression in the adult male. The others transcripts ARGONAUTE (sht_Ago_MS3_01142), DROSHA (sht_Drsh_MS3_06910) and DICER (sht_Dcr_MS3_05083 and sht_Dcr_MS3_09247), showed a low expression in all stages.

*Identification and characterisation of precursor and mature miRNAs in S. haematobium Egypt genome* - We identified in the *S. haematobium* Egypt genome 149 mature miRNAs and 131 pre-miRNAs [Supplementary data II **(Table I)**]. Regarding the miRNA precursor localisation in *S. haematobium* Egypt genome, 98 (73.68%) were found in intergenic regions and 34 (26.32%) intragenic [Supplementary data II **(Table II)**]. In addition, it was possible identify that 15 pre-miRNAs were organised in clusters (10kb as the maximum distance between two miRNAs genes to consider them clustered), such as sht-miR-71a/sht-miR-2a-1/sht-miR-2b/sht-miR-2e [Supplementary data I **(Fig. 2)**].

All pre-miRNAs identified were analysed for their structural and thermodynamic characteristics. The *S. haematobium* Egypt pre-miRNAs displayed MFE with an average of -25.3 kcal/mol, with values between -48.5 and -18.5 kcal/mol; AMFE values averaging -27.91 kcal/mol and MFEI with -0.752 kcal/mol [Supplementary data II **(Table III)**].

In addition, we performed statistical analyses using thermodynamic and structural characteristics of pre-miRNAs found in *S. haematobium* Egypt compared to pre-miRNAs deposited in the miRBase from Ecdysozoa, Cnidaria, Polifera and Schistosoma clades [Supplementary data II **(Table IV)**]. We compared the miRNA precursor characteristics from genus *Schistosoma* miRNA precursors and no differences were observed among the characteristics analysed (p > 0.05). On the other hand, in the analysis performed among *S. haematobium* Egypt against Ecdysozoa*,* Cnidaria and Porifera phylum, the pre-miRNA characteristics were significantly different (p < 0.05).

Results obtained using SRA data from male (SRR6655496) and female worms (SRR6655494) evidenced that all mature miRNAs were found in the genome, although some were not expressed in the libraries used for analysis. On the other hand, highly conserved miRNAs such as bantam, miR-2, let-7, miR-71, miR-125, miR-124 and miR-10 showed a greater amount of reads found in SRA data. In addition, some miRNAs were found only in the adult males library, suggesting possible targets for further studies [Supplementary data II **(Table V)**].

Of all precursor miRNAs, four families (miR-10, miR-8, miR-2 and miR-7) were selected to further analysis once they showed high conservation based on structural, thermodynamic characteristics and their phylogenetic distribution.

**Fig. 7:**
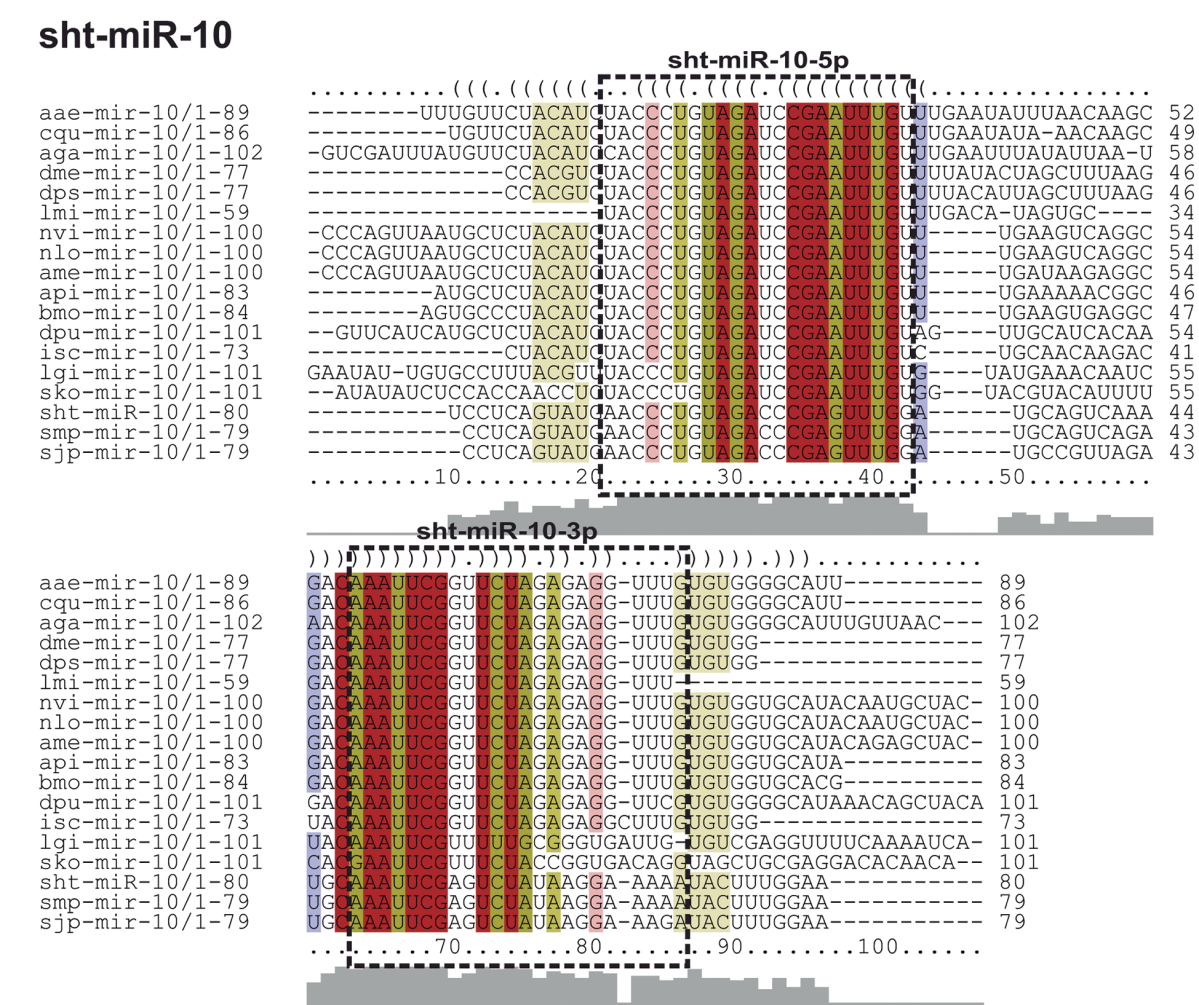
alignment of the sht-miR-10 pre-miRNA and their orthologs; sht: *Schistosoma haematobium*; smp: *S. mansoni*; sjp: *S. japonicum*; sko: *S. kowalevskii*; lgi: *Lottia gigantea*; isc: *Ixodes scapularis*; dpu: *Daphnia pulex*; bmo: *Bombyx mori*; api: *Acyrthosiphon pisum*; ame: *Apis mellifera*; nvi: *Nasonia vitripennis*; nlo: *Niphona longicornis*; lmi: *Locusta migratoria*; dps: *Drosophila pseudoobscura*; dme: *D. melanogaster*; aga: *Anopheles gambiae*; cqu: *Culex quinquefasciatus*; and aae: *Aedes aegypti*.

**Fig. 8:**
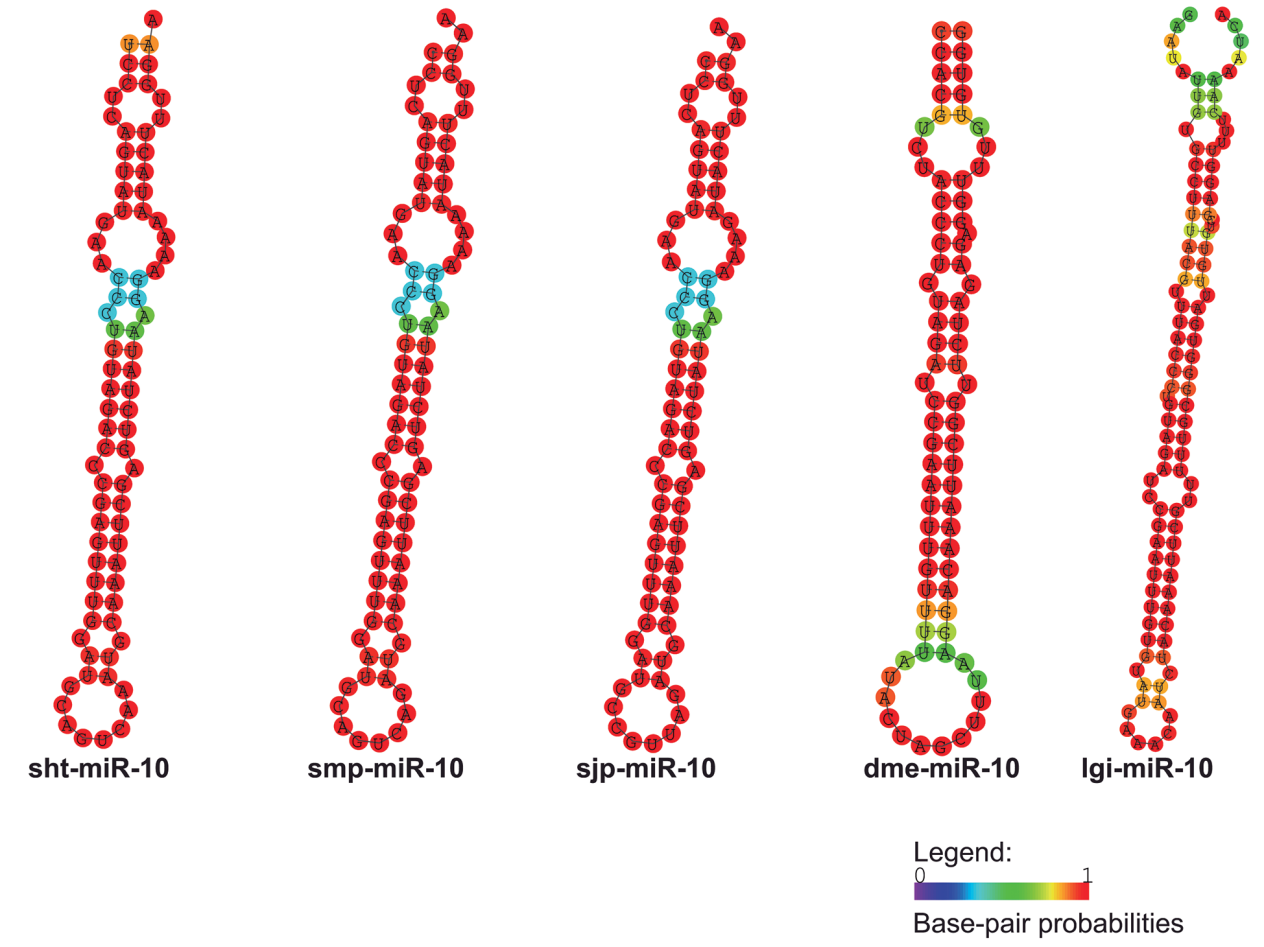
secondary structures of the sht-miR-10 pre-miRNA and their orthologs; sht: *Schistosoma haematobium*; smp: *S. mansoni*; sjp: *S. japonicum*, dme: *Drosophila melanogaster* and lgi: *Lottia gigantea.*

*sht-miR-10* - The sht-miR-10 precursor found in this study, showed two mature miRNAs, sht-miR-10-3p and sht-miR-10-5p. The sht-miR-10 precursor sequence presented a great conservation in the primary and secondary structures when compared to the ortholog sequences used for these analyses, as *S. mansoni*, *S. japonicum*, *Apis mellifera* and *D. melanogaster* (Figs 7, 8).

**Fig. 9:**
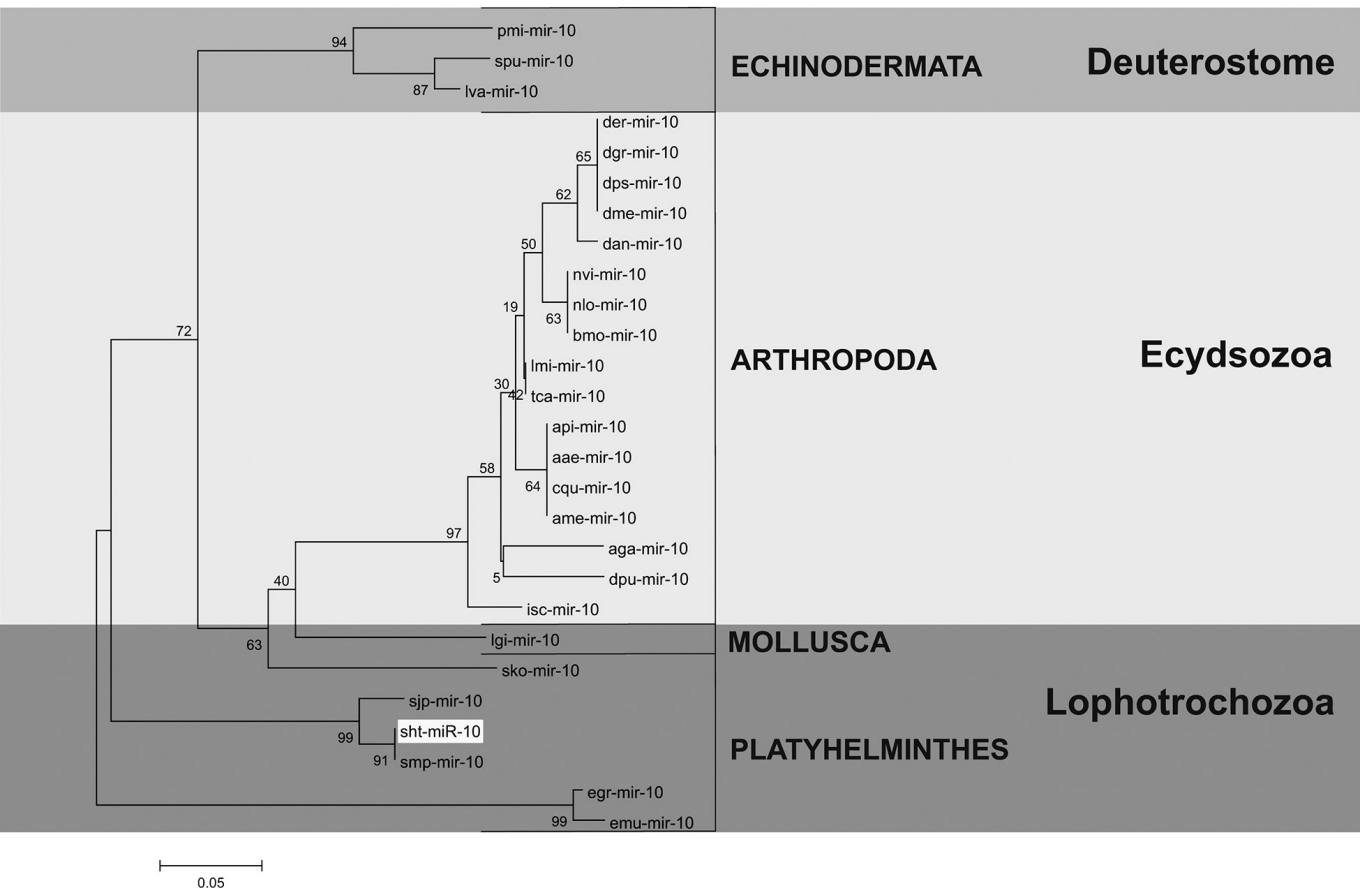
phylogenetic tree performed to sht-miR-10 pre-miRNA identified in *Schistosoma haematobium* Egypt genome and their orthologs; sht: *S. haematobium*; smp: *S. mansoni*; sjp: *S. japonicum*; egr: *Echinococcus granulosus*; emu: *Echinococcus multilocularis*; lgi: *Lottia gigantean*; isc: *Ixodes scapularis*; dpu: *Daphnia pulex*; aga: *Anopheles gambiae*; ame: *Apis mellifera*; cqu: *Culex quinquefasciatus*; aae: *Aedes aegypti*; api: *Acyrthosiphon pisum*; tca: *Tribolium castaneum*; lmi: *Locusta migratoria*; bmo: *Bombyx mori*; nvi: *Nasonia vitripennis*; nlo: *Niphona longicornis*; dan: *Drosophila ananassae*; dme: *Drosophila melanogaster*; dgr: *D. grimshtwi*; dps: *D. pseudoobscura*; der: *D. erecta*; lva: *Lytechinus variegatus*; spu: *Strongylocentrotus purpuratus*; and pmi: *Patiria miniata.*

Three clades were observed in the tree generated by phylogenetic analysis. The first clade was identified as Lophotrochozoa, showing sequences of Mollusca and Platyhelminthes organisms. The second clade, called Ecdysozoa, was composed by arthropods organism sequences and the third clade presented Echinodermata organisms. This distribution showed similarity to the tree of life, where *S. haematobium* Egypt remained alongside others flatworms (Fig. 9).

*sht-miR-8* - One precursor of miR-8 family was identified in the *S. haematobium* Egypt genome, sht-miR-8. Two mature miRNAs from this precursor also were found and named sht-miR-8-3p and sht-miR-8-5p. These sequences presented high conservation in relation to their orthologs in *S. mansoni*, *S. japonicum*, *Bombix mori*, *Heliconius melpomene*, *Anopheles gambiae*, *Daphnia pulex*, *Nasonia vitripennis*, *Ixodes scapularis* and *Lottia gigantea*, both for analyses of primary structure and secondary structures [Supplementary data I **(Figs 3, 4)**].

The phylogenetic analysis generated a tree divided in two clades: Lophotrochozoa and Ecdysozoa. The *S. haematobium* Egypt sequences were found closer to the main orthologs species, *S. japonicum* and *S. mansoni*, in the clade Lophotrochozoa, as well as organisms belonging to the Mollusca phylum. For the Escysozoa clade, the arthropod organisms were the only representative of this group. According to the conservation of the pre-miRNA sequences of these species, this distribution relation is also observed in the animal’s tree of life [Supplementary data I **(Fig. 5)**].

*sht-miR-2* - In miR-2 family of *S. haematobium* Egypt were identified five precursor miRNAs (sht-miR-2a-1, sht-miR-2a-2, sht-miR-2b, sht-miR-2c and sht-miR-2e) and nine mature miRNAs: sht-miR-2a-1-3p, sht-miR-2a-1-5p, sht-miR-2a-2-3p, sht-miR-2b-3p, sht-miR-2b-5p, sht-miR-2c-3p, sht-miR-2c-5p, sht-miR-2e-3p and sht-miR-2e-5p.

The *S. haematobium* Egypt miRNAs from this family exhibited high conservation as mature miRNAs when compared with their orthologs in *S. mansoni* and *S. japonicum* [Supplementary data I **(Fig. 6)**]. It was possible to observe that there is a great similarity between the secondary structures of the pre-miRNAs within the same family, as well as between *S. haematobium* and *S. mansoni* [Supplementary data I **(Fig. 7)**].

The tree generated from the phylogenetic analysis of miR-2 family showed a distribution into two clades. The clade Lophotrochozoa was composed by the Mollusca, Annelida and Platyhelminthe phylum, while the Ecdysozoa clade presented Arthropod organisms as its representative. Besides that, there was a correct grouping between all precursors sequences of this family (sht-miR-2a-1, sht-miR-2a-2, sht-miR-2b, sht-miR-2c and sht-miR-2e) with their respective orthologs species, mainly *S. mansoni* and *S. japonicum* [Supplementary data I **(Fig. 8)**].

*sht-miR-7* - Two precursor miRNAs of miR-7 family were identified: sht-miR-7 and sht-miR-7b. Besides that, three mature miRNAs also were found (sht-miR-7-5p and sht-miR-7b-5p) in *S. haematobium* Egypt genome. In the primary structure results of miR-7 family were observed a high conservation between the nucleotide sequences of *S. haematobium* miRNAs and their orthologs [Supplementary data I **(Fig. 9)**]. The analysis performed for pre-miRNA secondary structures of miR-7 family also evidenced a great conservation between the *S. haematobium* miRNAs and their orthologous organisms [Supplementary data I **(Fig. 10)**].

Three well defined clades were observed in the phylogenetic analysis performed for sht-miR-7 family: the Deuterostome group and the Lophotrochozoa and Ecdysozoa clades formed the Protostome group. The first group was composed of Chordata and Hemichordata phylum, while the second group had the presence of flatworms and arthropod organisms. The species classification showed by this analysis corroborates with the distribution found in the animal tree of life [Supplementary data I **(Fig. 11)**].

## DISCUSSION

The first version of *S. haematobium* genome published by Young et al. provided the conditions to researchers to study the genome and we were able to apply this robust analysis to identify and characterise miRNAs, and the putative proteins involved in miRNA processing pathway in the genome of this human parasite.^16^ The miRNAs and proteins of their biosynthetic pathway were described in several organisms, including plants and animals. In animals, in the model organisms *D. melanogaster* and *C. elegans*, were found mature and precursor miRNAs, pathway proteins and miRNA target genes.^29^ ^,^ ^30^ In addition, in species from *Schistosoma* genus, such as *S. mansoni* and *S. japonicum*, also were identified these regulatory molecules,^10^ ^,^ ^31^ ^,^ ^32^ showing that there have been an evolutionary conservation of these molecules.

Stroehlein et al.^32^ described mature and precursor miRNAs in *S. haematobium* genome data, however, they did not mention the identification, characterisation and expression of the miRNA pathway genes in the organism under study, as observed in our results. In addition, we were able to identify conserved and novel miRNAs, being 149 mature miRNAs and 131 miRNA precursors. Stroehlein and colleagues found 36 conserved miRNAs and 53 new miRNAs, while we identified 41 conserved miRNAs in the *Schistosoma* genus and 108 conserved miRNAs in others animal species. Of all mature miRNAs identified in both studies, only 25 mature miRNAs were found in both studies. 124 novel conserved mature miRNAs were found in this study comparing to Stroehlein and colleagues work. We also have validated the presence of the mature *S. haematobium* miRNAs using public smallRNA-seq libraries of male and female *S. haematobium* parasites.^32^

In miRNA processing and maturation, the proteins ARGONAUTE, DICER and DROSHA play a several key roles. In the nucleus, DROSHA complex protein is responsible for the first step in this pathway. The pri-miRNA is the product of the transcription characterised mainly by the formation of a stem-loop. This structure is targeted by DROSHA complex, that acting by the cleavage generate the pre-miRNAs.^33^ Protein DICER cleaves the pre-miRNAs removing the loop from the structure forming a miRNA duplex. In the RISC complex, DICER protein guides the miRNA duplex to ARGONAUTE proteins which one of the mature miRNA in the duplex target the target gene. A lot of others proteins are part of the RISC complex such as Fmr1, Tsn, VIG, among others.^34^

The family of ARGONAUTE proteins has two important subfamilies, AGO and PIWI. Both subfamilies play important roles in noncoding RNA pathways, being the AGO proteins the main protein of the RISC complex in the microRNA and small interfering RNA (siRNA) pathways, while PIWI proteins is responsible for specific RISC complex in PIWI-interecting RNAs (piRNA) pathway.^35^ This both ARGONAUTE subfamilies have shown two highly conserved domains, PIWI and PAZ domains. The PAZ domain has been presented at the N-terminal of the protein, while the C-terminal region has been composed by PIWI domain. The positions of theses domains are strategic, since they have the ability to position the small RNA in its target gene.^36^ ^,^ ^37^ The PIWI domain, in particular, has a catalytic site responsible for cleaving target messenger RNA (mRNA) of the miRNA. This active site has been composed by three amino acid residues: DDH (aspartate, aspartate and histidine) or DDD (three aspartates).^36^

All of these aspects in our analysis had a great importance. The putative ARGONAUTE genes found in *S. haematobium* Egypt showed the conserved domains, PAZ and PIWI. The phylogenetic tree showed two distinct clades. One clade showed only AGO orthologue proteins including sht_Ago_MS3_08447 and sht_Ago_MS3_0114 suggesting that these sequences are ARGONAUTE proteins. Furthermore, the catalytic site was identified in the PIWI domain of the *S. haematobium* Egypt ARGONAUTE proteins (DDH) corroborating with PIWI domain catalytic site reported by the literature. These proteins are considered one of the most important proteins involved in the miRNAs pathway, because they are catalytic component of RISC complex and help in the high silencing capacity of the miRNAs.^38^ ^,^ ^39^

All conserved domains described for the DICER and DROSHA proteins were found in the analyses of this work, where DROSHA showed the same conserved domains reported by others studies and DICER presented, besides the mentioned domains for this protein, one Dicer Dimer domain. It is important to emphasise that this analysis was possible once we used the new gene prediction beyond the predicted proteome from SchistoDB. We were able to improve the prediction of putative protein *S. haematobium* DROSHA. The presence of these domains found in *S. haematobium* Egypt DICER and DROSHA confirm that these proteins are ortholog of DICER and DROSHA. The DICER protein contains three main conserved domains, one PAZ domain and two domains called RNase III.^15^ ^,^ ^39^ In *S. mansoni*, in the sequences identified as DROSHA, were found three conserved domains: two RNase III and one DSRM domain.^15^

In addition, the analyses performed to identify the active site of two RNase III domains of DICER showed the distribution of following amino acids: glutamic acid (E), aspartate (D), aspartate (D) and glutamic acid (E), forming the site EDDE also present in *S. mansoni*.^15^ These findings found in DICER, DROSHA and AGO-like proteins of *S. haematobium* corroborate with the literature in domain distribution, conservation of active site and phylogenetic distribution confirming Thais prediction within *S. haematobium* data.

In the expression analysis using RNAseq data ― SRR6655495, SRR6655497, SRR6655493 ― the *S. haematobium* transcripts of ARGONAUTE, DROSHA and DICER showed in general low expression level in all three stages of development analysed. The highest expression way round for the VIG gene explained by the importance of this gene not only in the RISC complex of miRNA pathway but also in other mechanisms in the parasite such as the formation and maintenance of heterochromatin.^40^

24 mature miRNA sequences identified corroborated with Stroehlein et al.^32^ study such as bantam, let-7, miR-124 and others. Some studies involving *S. japonicum* were performed to identify miRNAs based on computational and experimental analysis. Xue et al.^31^ described five novel miRNAs while Huang et al. found 176 mature miRNAs using computational analyses.^41^ In *S. mansoni* 67 mature miRNAs and 42 precursor miRNAs were identified by Gomes et al.^10^ and 11 mature miRNAs were confirmed by experimental analyses by Simões et al.^42^ A comparison between *S. mansoni* and *S. haematobium* Egypt data showed that among the conserved miRNAs found in *S. mansoni*, only five of them were not identified in *S. haematobium* Egypt genome data in our study.

Most of the miRNA precursor genes was found in the intergenic regions of the *S. haematobium* Egypt genome [Supplementary data II **(Table II)**] corroborating with most animal species. We found 15 miRNAs in clusters. MiRNAs distributed in clusters has also been showed in other organisms such as in *S. japonicum*, *S. mansoni* and *B. glabrata*.^10^ ^,^ ^41^ ^,^ ^43^ ^,^ ^44^ ^,^ ^45^ The miRNA cluster gene mir-71/2a-1/2b/2e found in this work [Supplementary data I **(Fig. 2)**] has been reported in other organisms such as *S. mansoni*.^10^ The miRNA antiparallel clusters had been also occasionally found in other animals genomes deposited in miRBase version 22 (http://www.mirbase.org/), such as smp-miR-124b and smp-miR-124a from *S. mansoni*, mmu-miR-124-2 and mmu-miR-124b from *Mus musculus*, and oni-miR-124a-3 and oni-miR-124b-1 from *Oreochromis niloticus,* corroborating to our results [Supplementary data II **(Table II)**]. 

The pre-miRNAs sequences of *S. haematobium* Egypt displayed a Minimal Energy Free (MFE) with a mean of -25.3 kcal/mol, with values between -48.5 and -18.5 kcal/mol, and, considering the structural and thermodynamic characteristics already reported for miRNAs, a precursor miRNA molecule is stable and can generate mature miRNA having a value of the MFE of -20 kcal/mol. Besides that, this data corroborating with the values found in others animals studies, beyond corresponding to the values used for the orthologs *S. mansoni* and *S. japonicum*.^10^ ^,^ ^42^ The pre-miRNA size (~70nt) of *S. haematobium* miRNAs corroborated to orthologous size, indicate a high probability that these sequences are real pre-miRNA sequences.

In addition, the guanine-cytosine (GC) content found in the results of this work showed a distribution between 21.28% and 45.94% of GC content, but the majority of the sequences presented a percentage greater than 25%. This is one of the main parameters for pre-miRNAs identification and the presence of these nucleotides is very important for the stability of pre-miRNA secondary structure. In animals, evaluation value of this parameter should be found between 20% and 55%. As showed, the results indicated that the sequences analysed satisfy the requirements to be considered miRNA precursors. Previous works have shown how important has been the use of these structural and thermodynamic characteristics for conserved miRNA elucidation and prediction in the genome.^10^

The statistical analyses using thermodynamic and structural characteristics of the *S. haematobium* Egypt pre-miRNAs, when comparing the genus *Schistosoma* showed no differences between the groups (p > 0.05). Both displayed high conservation and similarity among pre-miRNAs from *S. haematobium* Egypt and species belonging to the genus *Schistosoma*. However, the results for *S. haematobium* Egypt were significantly different from results for other clades such as Ecdysozoa, Cnidaria and Porifera (p < 0.05), suggesting an evolutionary conservation of the pre-miRNA characteristics within each animals clade (superphylum, phylum or genus) and a significant difference among different clades [Supplementary data II **(Table IV)**]. The studies based on specific characteristics of mature and precursor miRNAs have been used in sequence groups in different species, from Protista to Metazoa and Plantae.^11^ ^,^ ^46^ ^,^ ^47^ ^,^ ^48^

Our analyses using the libraries of small RNAs from male and female adult worms showed a validation of all 149 mature miRNAs found in the genome of *S. haematobium* Egypt [Supplementary data II **(Table V)**]. Some conserved miRNAs such as bantam-3p, let-7-3p, miR-124-3p and miR-10-5p demonstrated a large number of reads in two libraries studied, corroborating with the results found in a previously published study,^32^ however, our findings refer to mature miRNAs. Other miRNAs have also identified in both studies, such as the miRNAs of the miR-2, miR-7 and miR-8 families demonstrating the importance of these molecules in both libraries, female and male adult worms.^32^

The miR-10 was found in *S. haematobium* Egypt genome containing two mature miRNAs (sht-miR-10-5p and sht-miR-10-3p), corroborating with results observed in studies with *D. melanogaster* and *S. japonicum*.^43^ ^,^ ^49^ Besides that, when compared to the pre-miR-10 sequences of *S. mansoni*, *S. haematobium* Egypt and *S. japonicum* had 100% identity in the region of their two mature miRNAs (Fig. 7). Many different animal species have shown this miRNA and its presence in the HOX cluster, composed by miR-10 and HOX genes. This relationship was observed in organisms such as *S. mansoni*, *A. gambiae*, *Tribolium castaneum*, zebrafish and human.^10^ ^,^ ^50^ On the other hand, miR-10 was one of five miRNAs considered pathogen-specific found in rabbits infected with *S. japonicum*, which may offer a new biomarker to diagnose schistosomiasis.^51^

The results miR-8 precursor shown two mature miRNAs. Besides that, in *S. haematobium* a high conservation was observed among the species belonging to Lophotrochozoa and Ecdysozoa clades, being considered a Protostome-specific miRNA, corroborating with *S. mansoni* data.^52^ This miRNA was found in several organisms, including the model organism *Drosophila* sp. that was also identified by computational analyses.^49^ In another study, miR-8 was identified in two mosquito’s vector: *Aedes albopictus* and *Culex quinquefasciatus*.^53^ In *S. japonicum*, miR-8 also was identified and presented a greater expression in mature adult worms, but was observed with some nucleotide substitutions considered specific for *Schistosoma* sp.^43^ MiR-8-3p was found 11 times more reads in the male library compared to the amount of reads found in the female library which were 104 reads. For mature miRNA miR-8-5p, none read was found in the male library and in the female library.

The miR-2 family showed five precursors and ten mature miRNA in *S. haematobium* data. This family is specific of protostome organisms, since in the phylogenetic tree were found only organisms from Ecdysozoa and Lophotrochozoa clades, corroborating with the results shown by Gomes et al.^52^. Besides that, miR-2 family has considered one of the larger miRNA family found in *D. melanogaster* presenting eight miRNAs in the genome.^54^ On the other hand, in *C. elegans*, another model organism, there is only one sequence of this family.^55^ In *S. mansoni*, nine members belonging to the miR-2 family were identified and some were in the cluster with miR-71.^10^ This cluster is located on a sexual chromosome in the parasite and therefore, may play a role in their sexual maturation.^44^

The sequences of miR-7 family corroborated with miRNAs found in *S. mansoni*. In addition, the secondary structures performed for miR-7b, in both studies, show a high conservation of these sequences.^52^ This family is one of the most conserved miRNA families and was found in all Bilateria, including flatworms, nematodes, insects and vertebrates.^56^ In *S. japonicum*, miR-7 was more expressed in male worms than in females; on the other hand, this miRNA is expressed in a specific stage of parasite life, mainly in cercariae stage. Data involving the validation of these miRNAs showed that in the small RNA library of adult male there were more reads of miR-7 compared to adult female library. The mature miRNA miR-7-5p demonstrates 25 times more reads in males library compared to females library. The miR-7 is related with different cellular processes. In *Drosophila* sp., it was reported its involvement in the formation of photoreceptor organs^57^ and in a study with rat pancreas was observed that miR-7 is the miRNA most abundant in this cell type, confirming the performance flexibility of this miRNA.^58^

According to the results obtained in this work, through bioinformatics and computational analyses we were able to identify 14 key proteins involved in small RNA processing pathway. Our results showed that the miRNA pathway genes were transcriptionally active. In addition, we identified 149 mature miRNAs and 131 pre-miRNAs in the genome of this species, being highly conserved in their sequences, corroborating with others studies involving *S. mansoni* and *S. japonicum*. The results found in this work will open up a new avenue for studying the miRNAs in the *Schistosome* biology and technologies involving the gene silencing control in this organism. In addition, will allow understanding many processes and mechanisms in their biology, since these molecules present a significant regulatory role for different mRNAs, affecting various characteristics of the organism in different stages and environmental conditions.
