# Supplementary material for: Computational prediction and characterisation of miRNAs and their pathway genes in human schistosomiasis caused by Schistosoma haematobium
Source: Mem Inst Oswaldo Cruz. 2020 May 8;115:e190378. doi: 10.1590/0074-02760190378 (PMC7207159; doi:10.1590/0074-02760190378)
Supplement: Supplementary file 1 [file 1678-8060-mioc-115-e190378-s1.pdf]

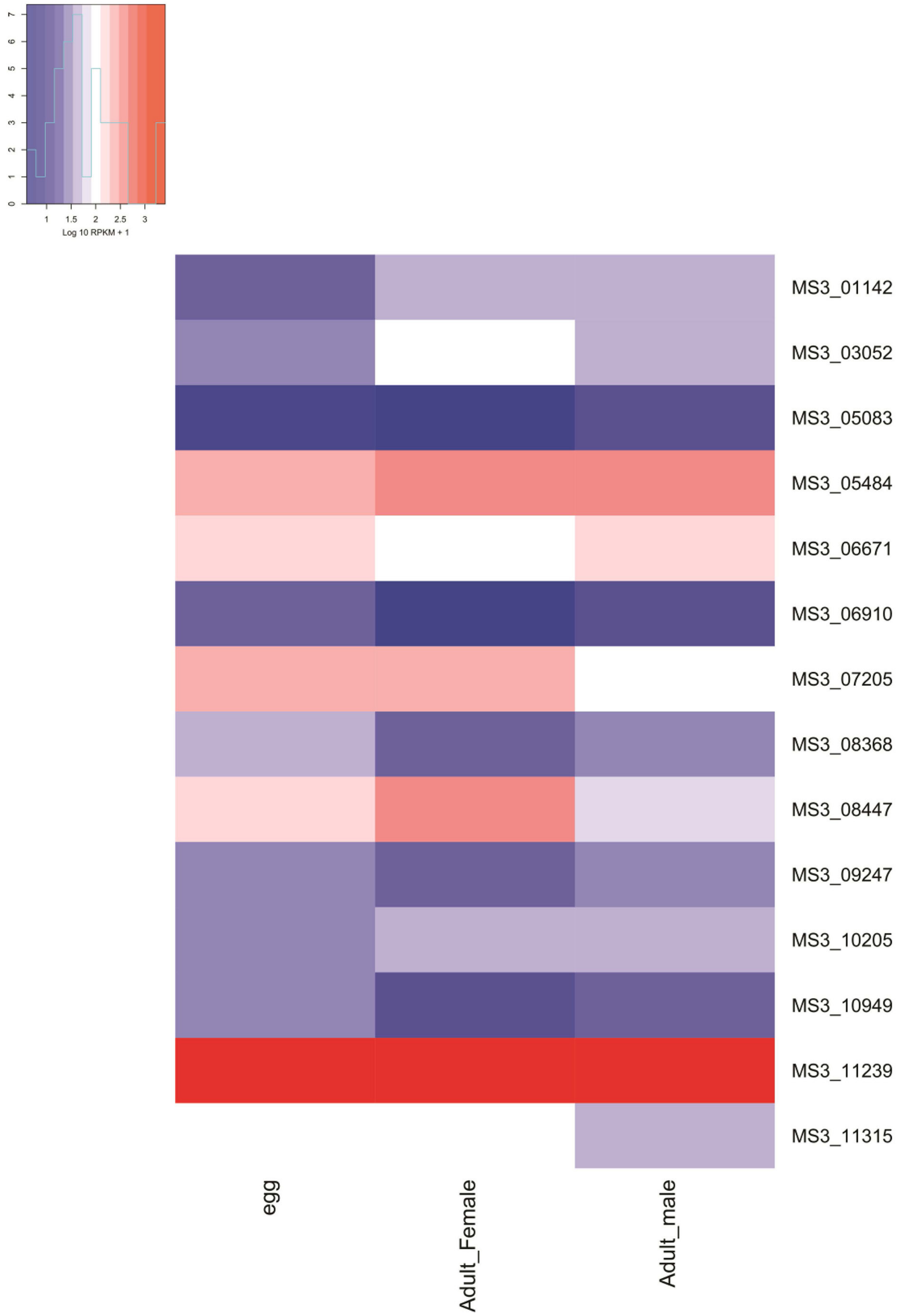

Fig. 1: expression profile of genes encoding the proteins involved in the miRNA pathway found in *Schistosoma haematobium* Egypt.

CC BY

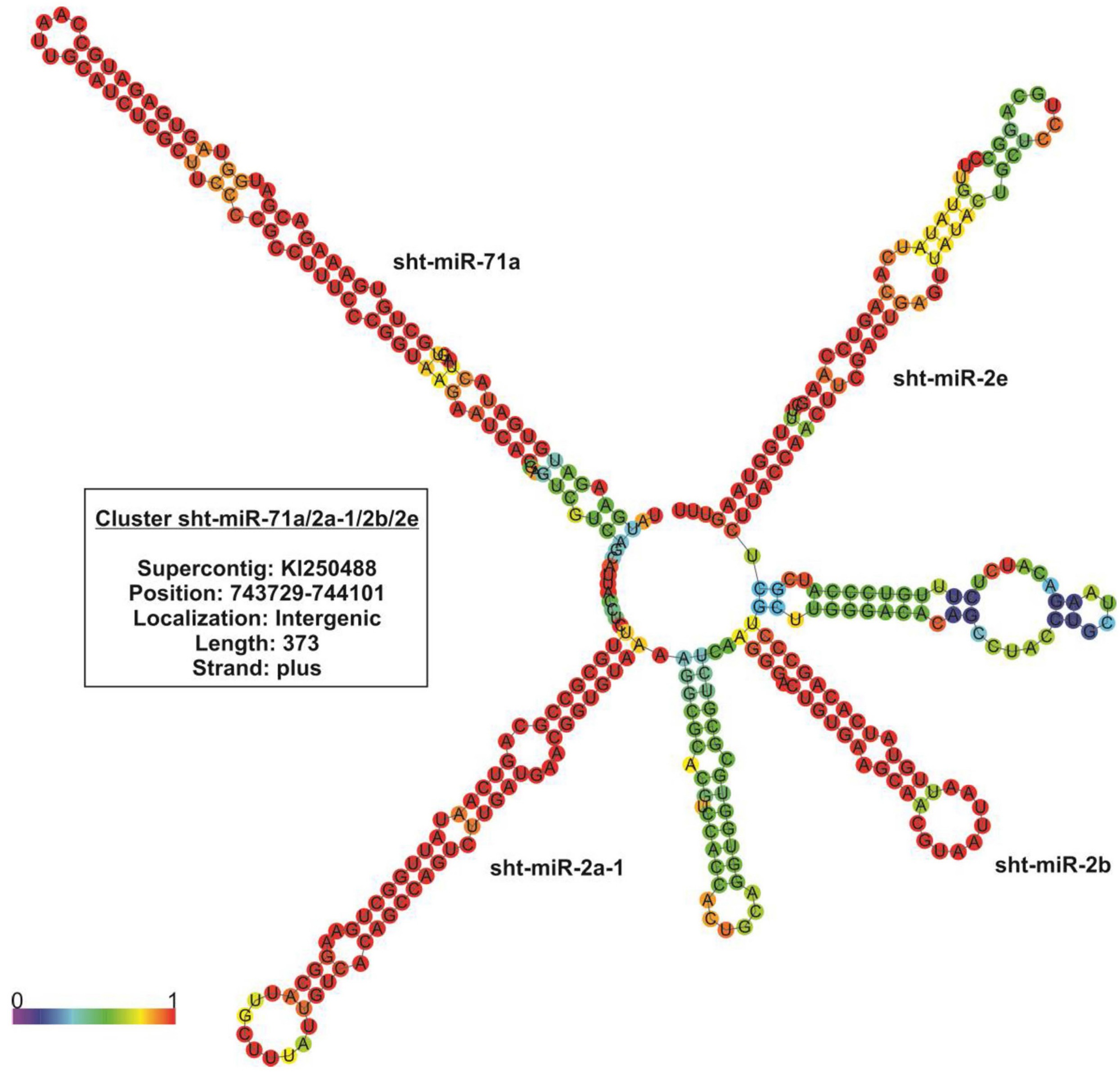

Fig. 2: RNA secondary structure of the cluster sht-miR-71a/sht-miR-2a-1/sht-miR-2b/sht-miR-2e; sht: *Schistosoma haematobium*.

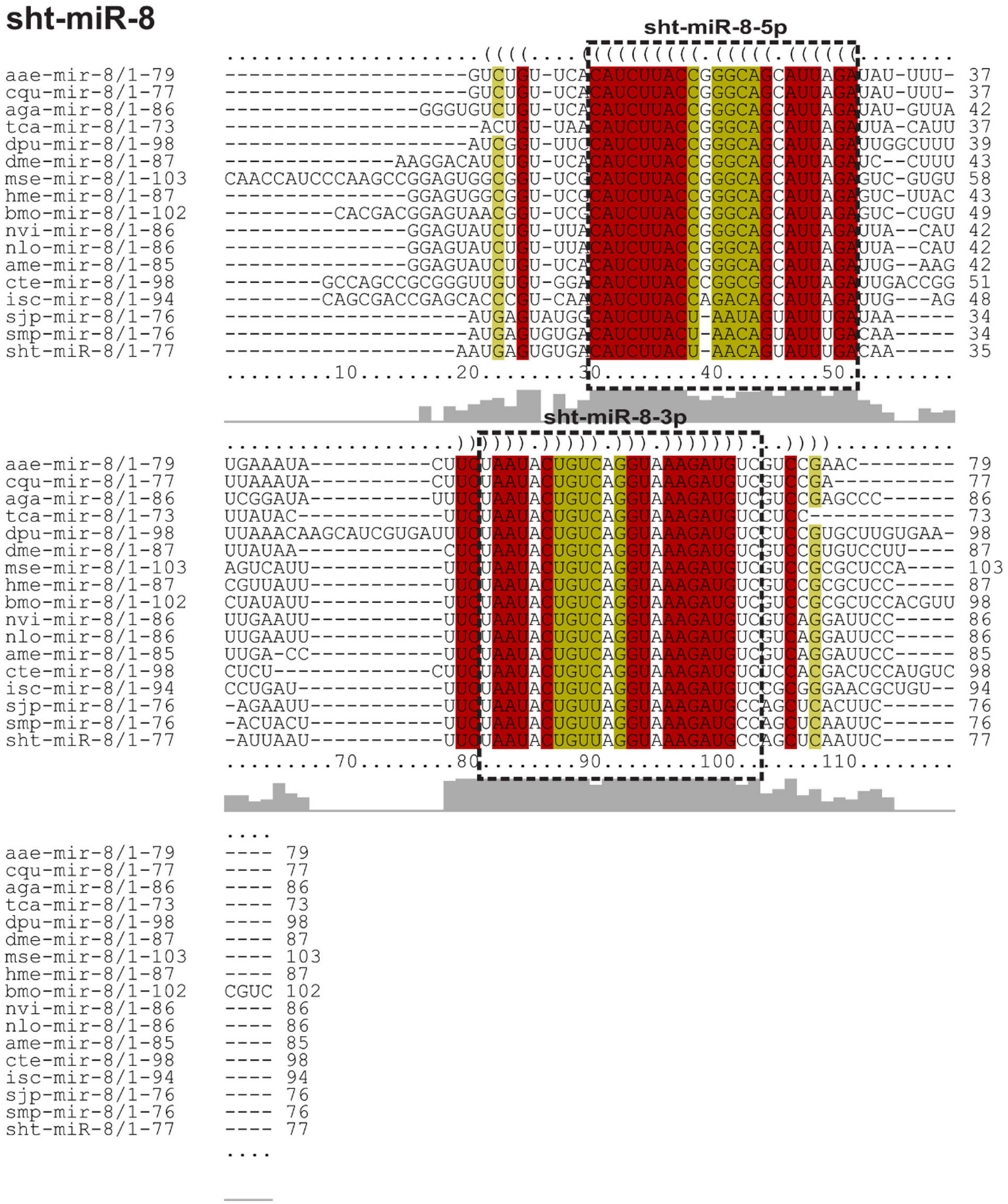

Fig. 3: alignment of sht-miR-8 pre-miRNA and their orthologs; sht: *Schistosoma haematobium*; smp: *S. mansoni*; sjp: *S. japonicum*; isc: *Ixodes scapularis*; cte: *Capitella teleta*; ame: *Apis mellifera*; nlo: *Nasonia longicornis*; nvi: *Nasonia vitripennis*; bmo: *Bombix mori*; hme: *Heliconius melpomene*; mse: *Manduca sexta*; dme: *Drosophila melanogaster*; dpu: *Daphnia pulex*; tca: *Tribolium castaneum*; aga: *Anopheles gambiae*; cqu: *Culex quinquefasciatus*; and aae: *Aedes aegypti*.

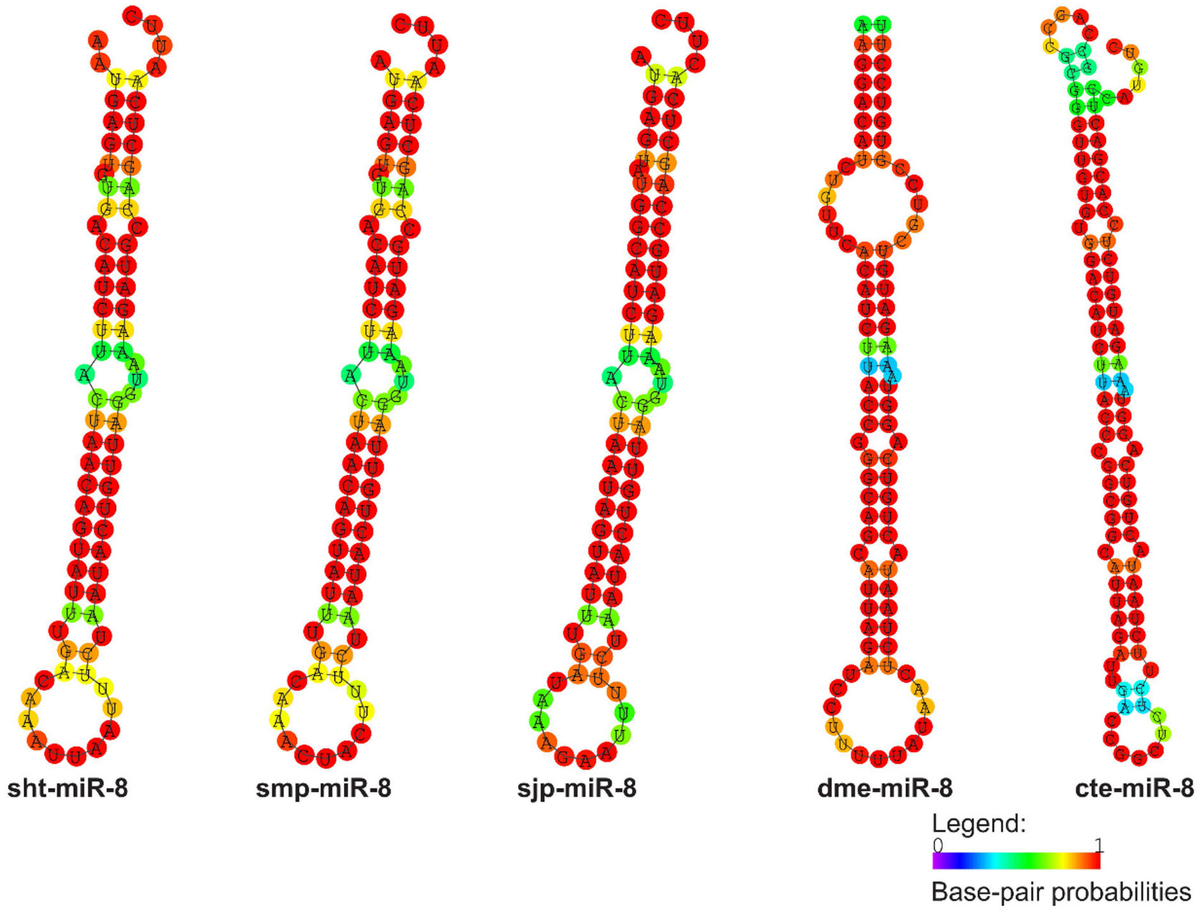

Fig. 4: secondary structures of the sht-miR-8 pre-miRNA and their orthologs; sht: *Schistosoma haematobium*; smp: *S. mansoni*; sjp: *S. japonicum*; dme: *Drosophila melanogaster*; and cte: *Capitella teleta*.

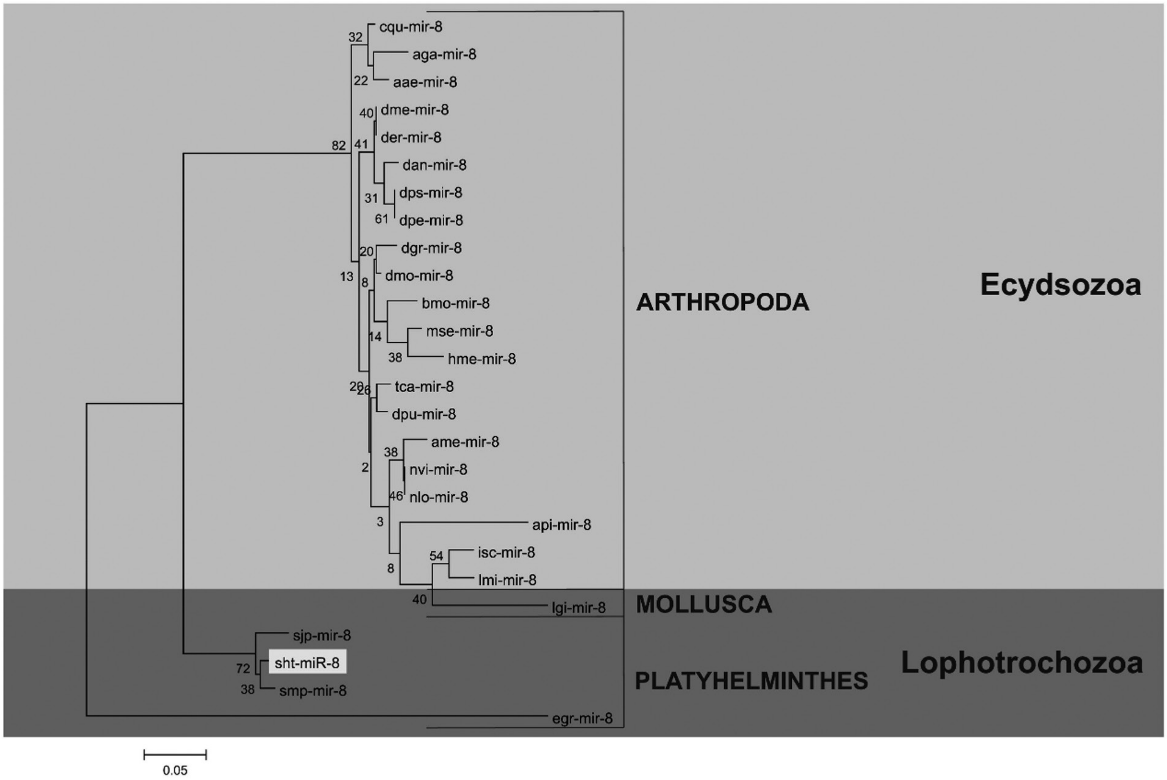

Fig. 5: phylogenetic tree generated by phylogenetic analysis to sht-miR-8 precursor miRNAs found in *Schistosoma haematobium* Egypt and their orthologs; sht: *S. haematobium*; smp: *S. mansoni*; sjp: *S. japonicum*; egr: *Echinococcus granulosus*; lgi: *Lottia gigantean*; lmi: *Locusta* migratoria; isc: *Ixodes scapularis*; api: *Acyrthosiphon pisum*; nlo: *Nasonia longicornis*; nvi: *Nasonia vitripennis*; ame: *Apis mellifera*; dpu: *Daphnia pulex*; tca: *Tribolium castaneum*; hme: *Heliconius Melpomene*; mse: *Manduca sexta*; bmo: *Bombyx mori*; dmo: *Drosophila mojavensis*; dgr: *Drosophila grimshtwi*; dpe: *Drosophila persimilis*; dps: *Drosophila pseudoobscura*; dan: *Drosophila ananassae*; der: *Drosophila erecta*; dme: *D. melanogaster*; aae: *Aedes aegypti*; aga: *Anopheles gambiae*; and cqu: *Culex quinquefasciatus*.

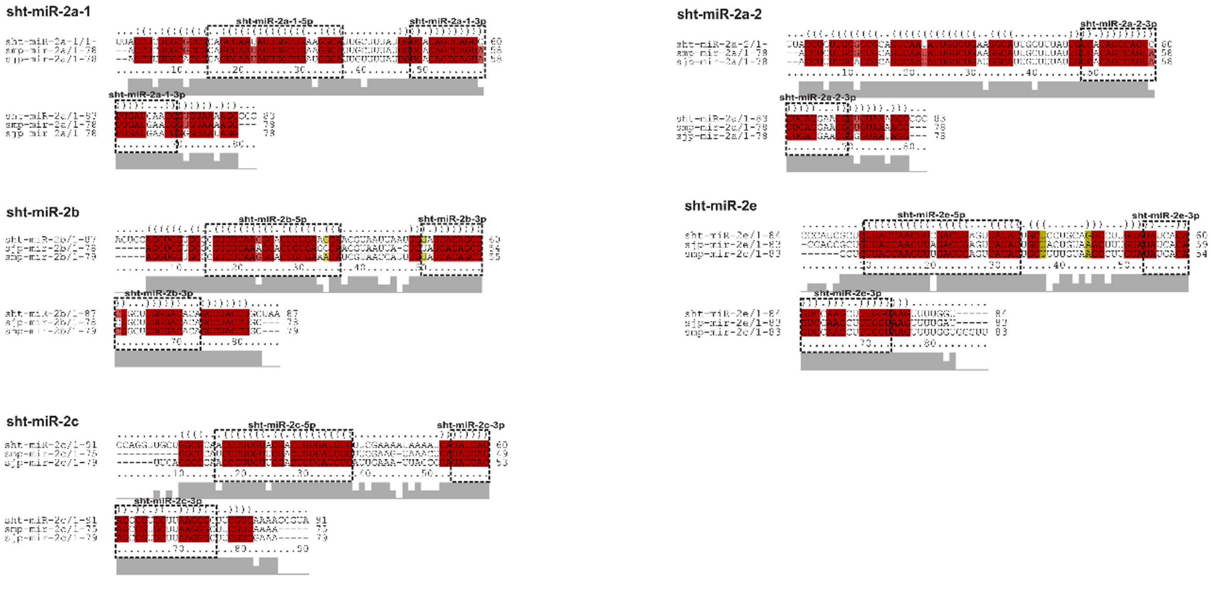

Fig. 6: alignment of the sht-miR-2 family pre-miRNAs and their orthologs; sht: *Schistosoma haematobium*; smp: *S. mansoni*; sjp: *S. japonicum*.

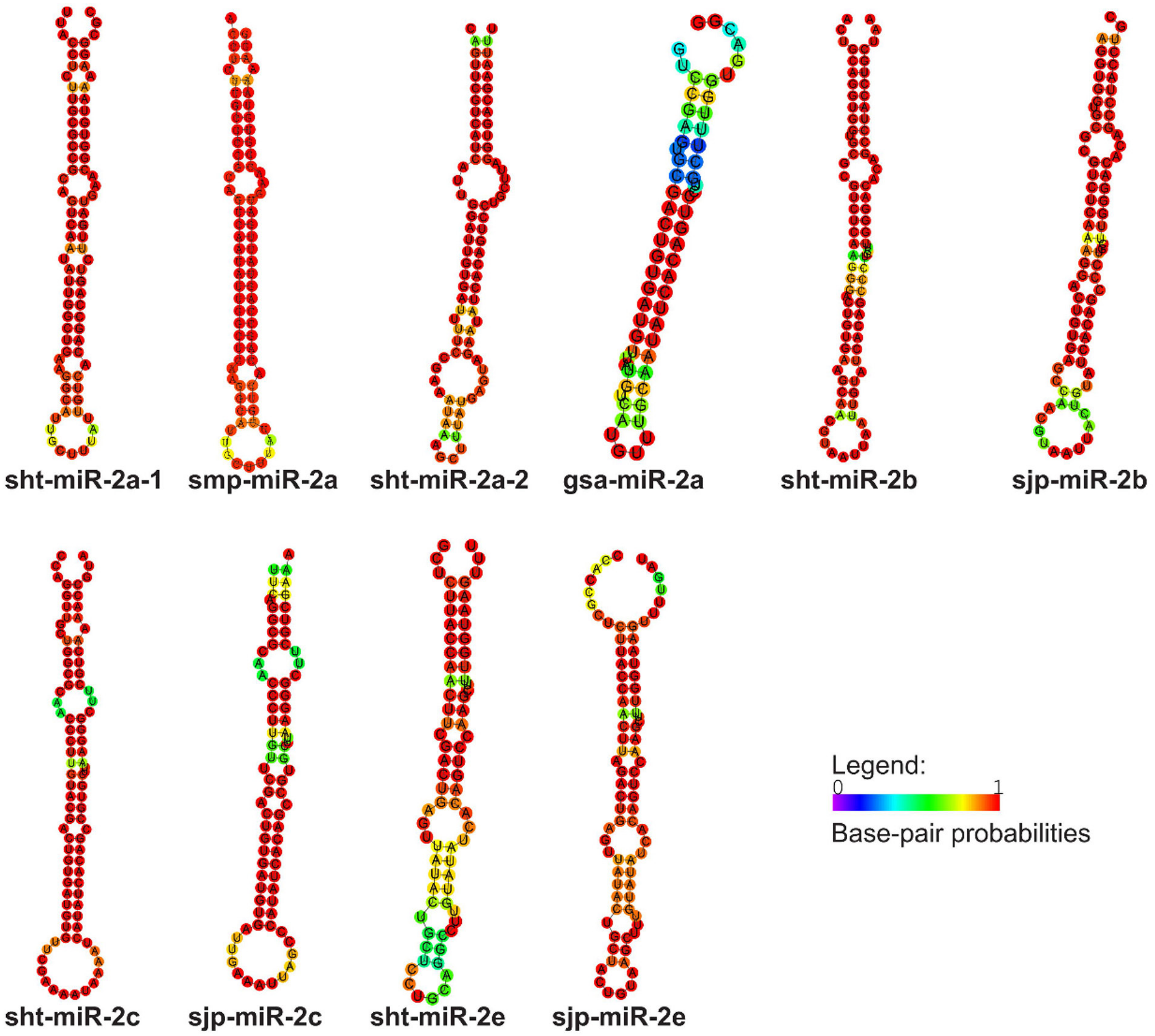

Fig. 7: secondary structures of the sht-miR-2 family pre-miRNAs and their orthologs; smp: *Schistosoma mansoni*; sht: *S. haematobium*; gsa: *Gyrodactylus salaris*.

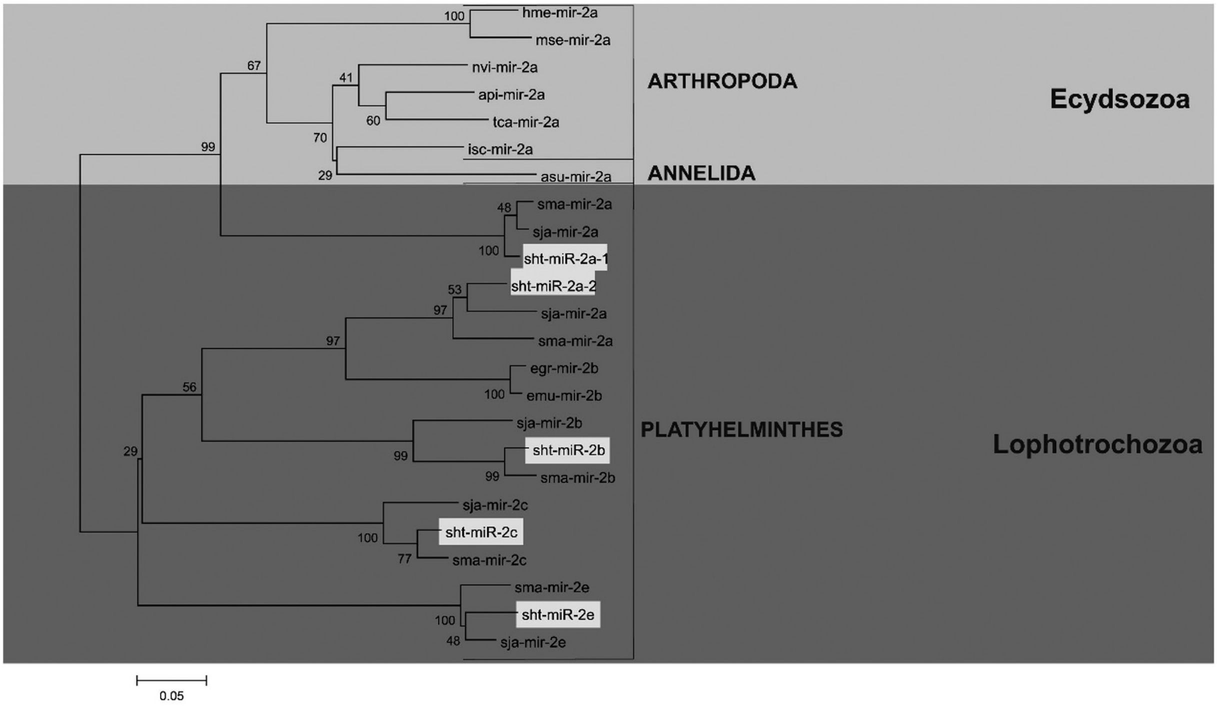

Fig. 8: tree generated from the phylogenetic analysis to sht-miR-2 family precursor miRNAs and their orthologs; sht: *Schistosoma haematobium*; smp: *S. mansoni*; sjp: *S. japonicum*; emu: *Echinococcus multilocularis*; egr: *Echinococcus granulosus*; asu: *Ascaris suum*; isc: *Ixodes scapularis*; tca: *Tribolium castaneum*; api: *Acyrthosiphon pisum*; nvi: *Nasonia vitripennis*; mse: *Manduca sexta*; and hme: *Heliconius melpomene.* Fig. 8: tree generated from the phylogenetic analysis to sht-miR-2 family precursor miRNAs and their orthologs; sht: *Schistosoma haematobium*; smp: *S. mansoni*; sjp: *S. japonicum*; emu: *Echinococcus multilocularis*; egr: *Echinococcus granulosus*; asu: *Ascaris suum*; isc: *Ixodes scapularis*; tca: *Tribolium castaneum*; api: *Acyrthosiphon pisum*; nvi: *Nasonia vitripennis*; mse: *Manduca sexta*; and hme: *Heliconius melpomene.*

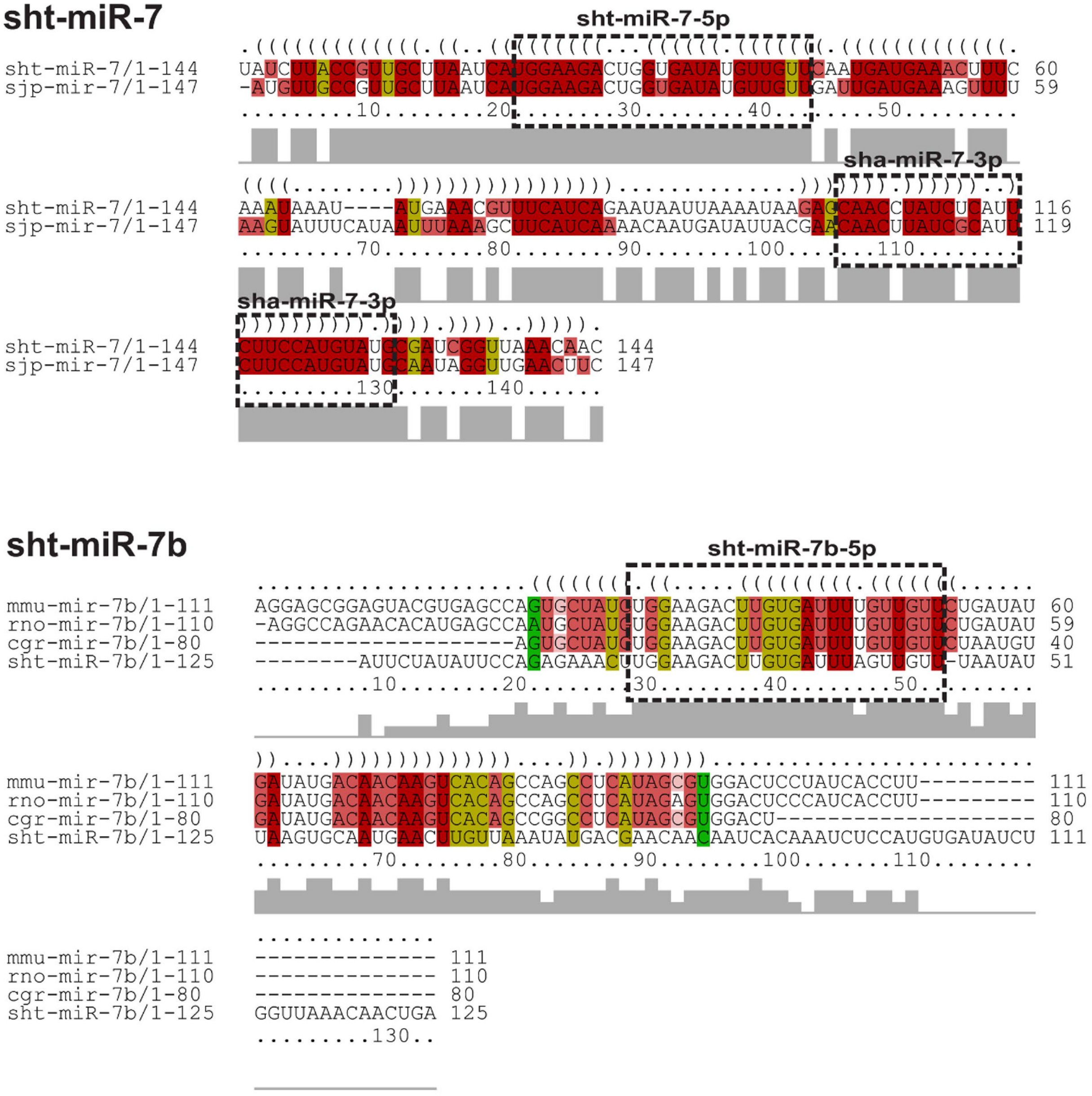

Fig. 9: alignment of the sht-miR-7 and sht-miR-7b sequences and their orthologs; sht: *Schistosoma haematobium*; sjp: *S. japonicum*; mmu: *Mus musculus*; rno: *Rattus norvegicus*; cgr: *Cricetulus griseus.*

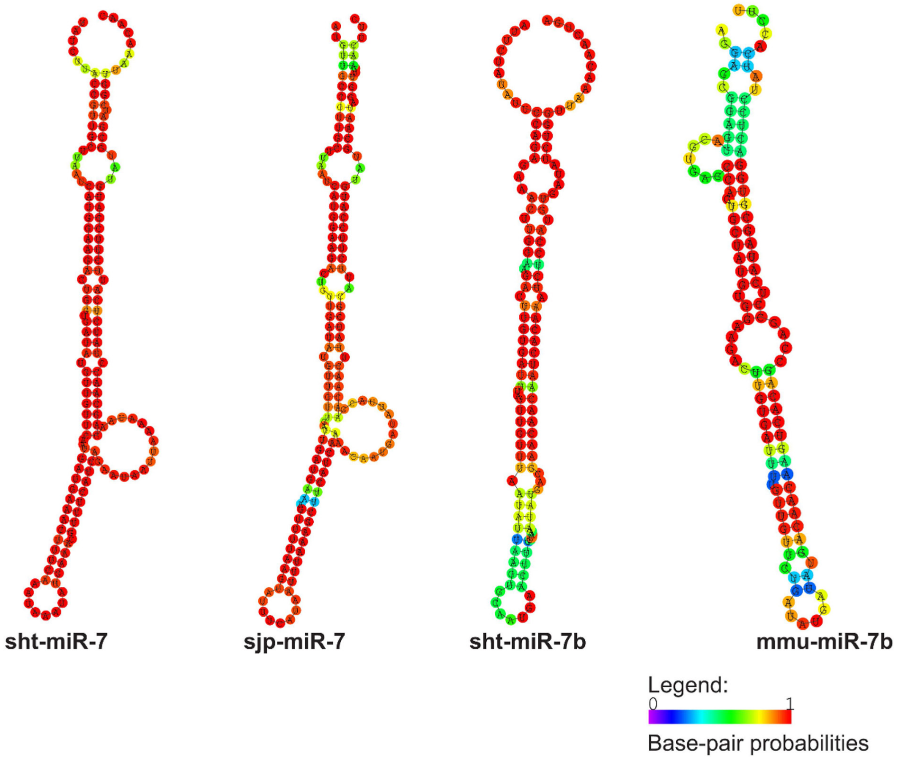

Fig. 10: secondary structures of the sht-mir-7 and sht-mir-7b pre-miRNAs and their orthologs; sht: *Schistosoma haematobium*; sjp: *S. japonicum*; mmu: *Mus musculus*.

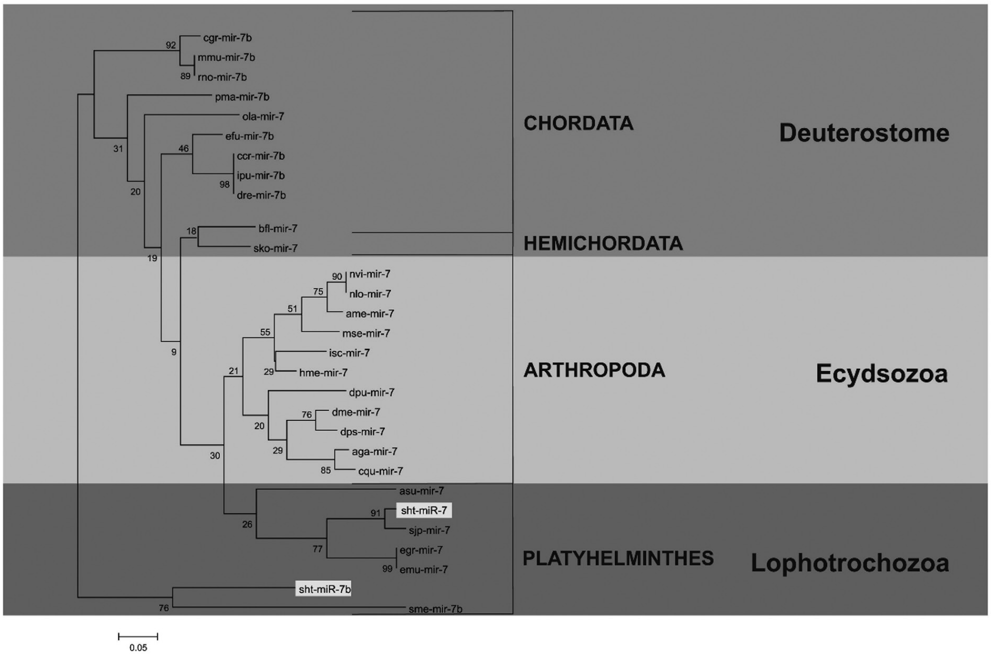

Fig. 11: phylogenetic tree of sht-miR-7 family precursor miRNAs and their orthologs; sht: *Schistosoma haematobium*; sjp: *S. japonicum*; sme: *S. mediterranea*; egr: *Echinococcus granulosus*; emu: *E. multilocularis*; asu: *Ascaris suum*; cqu: *Culex quinquefasciatus*; aga: *Anopheles gambiae*; dps: *Drosophila pseudoobscura*; dme: *D. melanogaster*; dpu: *Daphnia pulex*; hme: *Heliconius Melpomene*; isc: *Ixodes scapularis*; mse: *Manduca sexta*; ame: *Apis mellifera*; nlo: *Niphona longicornis*; nvi: *Nasonia vitripennis*; sko: *Saccoglossus kowalevskii*; bfl: *Branchiostoma floridae*; dre: *Danio rerio*; ipu: *Ictalurus punctatus*; ccr: *Cyprinus carpio*; efu: *Eptesicus fuscus*; ola: *Oryzias latipes*; pma: *Petromyzon marinus*; rno: *Rattus norvegicus*; mmu: *Mus musculus*; and cgr: *Cricetulus griseus*.
